# Reproducibility and robustness of high-throughput S1500+ transcriptomics on primary rat hepatocytes for chemical-induced hepatotoxicity assessment^☆^

**DOI:** 10.1016/j.crtox.2021.07.003

**Published:** 2021-08-05

**Authors:** Fan Lee, Imran Shah, Yun Ting Soong, Jiangwa Xing, Inn Chuan Ng, Farah Tasnim, Hanry Yu

**Affiliations:** a Innovations in Food & Chemical Safety Program (IFCS), Institute of Bioengineering and Bioimaging (IBB), Agency for Science Technology and Research, Singapore; b Center for Computational Toxicology & Exposure, Office of Research and Development, U.S. Environmental Protection Agency, Research Triangle Park, NC, United States; c Department of Physiology and Mechanobiology Institute, National University of Singapore, Singapore; d Critical Analytics for Manufacturing Personalized-Medicine, Singapore-MIT Alliance for Research and Technology, Singapore

**Keywords:** High-throughput transcriptomics, Hepatotoxicity, Rat hepatocytes, Collagen sandwich, TempO-Seq, S1500+ gene set, Gene set enrichment analysis, Hallmark gene set collection, Connectivity mapping

## Abstract

Cell-based *in vitro* models coupled with high-throughput transcriptomics (HTTr) are increasingly utilized as alternative methods to animal-based toxicity testing. Here, using a panel of 14 chemicals with different risks of human drug-induced liver injury (DILI) and two dosing concentrations, we evaluated an HTTr platform comprised of collagen sandwich primary rat hepatocyte culture and the TempO-Seq surrogate S1500+ (ST) assay. First, the HTTr platform was found to exhibit high reproducibility between technical and biological replicates (r greater than 0.85). Connectivity mapping analysis further demonstrated a high level of inter-platform reproducibility between TempO-Seq data and Affymetrix GeneChip data from the Open TG-GATES project. Second, the TempO-Seq ST assay was shown to be a robust surrogate to the whole transcriptome (WT) assay in capturing chemical-induced changes in gene expression, as evident from correlation analysis, PCA and unsupervised hierarchical clustering. Gene set enrichment analysis (GSEA) using the Hallmark gene set collection also demonstrated consistency in enrichment scores between ST and WT assays. Lastly, unsupervised hierarchical clustering of hallmark enrichment scores broadly divided the samples into hepatotoxic, intermediate, and non-hepatotoxic groups. Xenobiotic metabolism, bile acid metabolism, apoptosis, p53 pathway, and coagulation were found to be the key hallmarks driving the clustering. Taken together, our results established the reproducibility and performance of collagen sandwich culture in combination with TempO-Seq S1500+ assay, and demonstrated the utility of GSEA using the hallmark gene set collection to identify potential hepatotoxicants for further validation.

## Highlights

• TempO-Seq assays of rat hepatocytes in collagen sandwich are highly reproducible.
• Gene expression analysis shows S1500+ is representative of the whole transcriptome.
• Connectivity mapping shows consistency between TempO-Seq and Affymetrix data.
• Gene set enrichment shows consistency between S1500+ and the whole transcriptome.
• Gene set enrichment using hallmark gene sets informs hepatotoxicity.

## Graphical abstract

**Figure:**
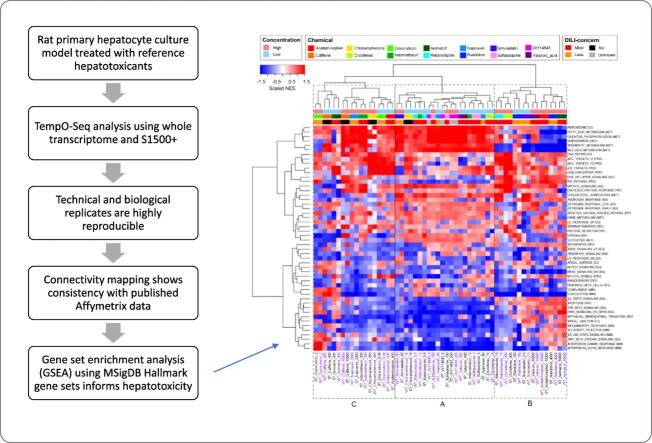

## Introduction

Acute or chronic exposure to drugs and environmental chemicals can result in hepatotoxicity, which is attributed in part to the liver generating reactive metabolites that interact with cellular macromolecules, disrupting their functions and leading to stress (Park et al., 2004, Sturgill and Lambert, 1997). Stress accumulated beyond the liver’s homeostatic capacity can cause necrosis, apoptosis, or autophagy, eventually resulting in liver diseases or even failure (Hinson et al., 2010, Iorga et al., 2017). Assessing the risks of chemical exposure to human health, including the liver, has traditionally been performed through characterizing their effects in animals, which is both resource and time-consuming. Animal-based toxicity testing also has ethical concerns due to the large number of animals required and the distress caused. To address the limitations of animal-based toxicity testing, we recognized a combination of *in vitro* high-throughput assays and computational modeling approaches as the way forward (Kavlock et al., 2018, Krewski et al., 2010).

Part of the vision of toxicity testing using *in vitro* assays is to identify perturbed molecular targets or biological pathways to associate chemicals with adverse outcome pathways (AOPs) (Krewski et al., 2010, Thomas et al., 2019). The U.S. EPA’s ToxCast program and the Tox21 consortium have utilized high-throughput assays to measure changes in biological pathways of relevance to toxicity. To date, the assays implemented in Tox21 cover an estimated 63% of the 1658 distinct human biological pathways (Huang et al., 2019). To broaden the coverage, high-throughput transcriptomics (HTTr) was selected as one of the first tier high-throughput assays for capturing potential chemical hazards in a tiered testing framework for chemical hazard characterization described by the U.S. EPA (Harrill et al., 2019; Russell S Thomas et al., 2019). To increase the throughput and reduce the cost of HTTr data acquisition, a set of approximately 2700 unique genes, called the S1500+ gene set, has been developed as a surrogate to the whole transcriptome (Mav et al., 2020, Mav et al., 2018). Combined with a targeted sequencing platform known as TempO-Seq® (Yeakley et al., 2017), the surrogate S1500+ gene set (Yeakley et al., 2017) has demonstrated comparable performance to established whole transcriptome techniques such as microarray and RNA-Seq in capturing chemical-induced gene expression changes in rat liver (Bushel et al., 2018). TempO-Seq data of differentiated HepaRG cultures has also been shown to be capable of identifying liver injury compounds using benchmark concentration modeling and relevant biological-response pathways using Ingenuity Pathway Analysis (Ramaiahgari et al., 2019).

Gene set enrichment analysis (GSEA) is a powerful method for interpreting genome-wide expression profiles by grouping individual genes into gene sets that represent a specific biological state or process sharing the same chromosomal location or regulatory targets (Subramanian et al., 2005). GSEA is frequently used together with the Molecular Signature Database (MSigDB), which contains over 25,000 gene sets divided into 8 major collections. Notably, the hallmark collection – 50 gene sets that each represent a well-defined biological state or process – offers a concise way of summarizing chemical-induced changes in gene expression (Liberzon et al., 2015). Indeed, GSEA using the hallmark gene set collection has been shown to be capable of detecting chemical- and dose-specific, as well as transient and sustained patterns of transcriptional enrichment in microarray data of rat liver (Dean et al., 2017). Analysis of microarray data from the Open TG-GATEs database by GSEA using gene sets derived from the Reactome pathway database and biclustering has also revealed conserved patterns of chemical-induced transcriptional responses in rat liver and rat and human hepatocytes (El-Hachem et al., 2016).

Although the S1500+ gene set and the hallmark gene set collection were independently developed, both aim to reduce redundancy without compromising on the coverage of biological space. The former achieves this at the gene level and the latter at the gene set level. We reasoned that GSEA of S1500+ genes using the hallmark gene set collection would inform the effects of a chemical on the liver and provide a common pattern of enrichment that represents hepatotoxicity. However, GSEA was originally designed to run on the entire transcriptome (Subramanian 2005), and studies comparing GSEA of the whole transcriptome to that of a subset of genes representing the whole transcriptome are limited. To examine if the S1500+ gene set could produce comparable results as the whole transcriptome in GSEA, we selected sandwich cultured rat hepatocytes as a resource-sparing and scalable model for hepatotoxicity screening, measured gene expression using the TempO-Seq ST HTTr assay, and performed GSEA with the hallmark gene set collection. We chose the collagen sandwich culture configuration amongst a plethora of 2D/3D cultures for balanced operational simplicity/robustness and maintenance of hepatocyte functions for up to a week (De Bruyn et al., 2013, Dunn et al., 1991), and limited chemical treatment to 24 h to achieve a rapid turnaround time and sufficient perturbations in cultured hepatocytes to induce chemical-specific gene signatures (Igarashi et al., 2014).

Since establishing the performance of a high-throughput platform is essential before incorporating the data for chemical risk assessment, the goals of this study were to (1) investigate the intra- and inter-batch reproducibility of the HTTr platform, as well as concordance with external data, (2), compare the TempO-Seq surrogate S1500+ transcriptome (ST) assay with the whole transcriptome (WT) assay in detecting chemical-induced changes at the gene and pathway levels, and (3) identify a general pattern of hallmark gene set enrichment that represents hepatotoxicity. Our results established the performance and reproducibility of the HTTr platform and demonstrated the potential of combining S1500+ high-throughput transcriptomics with hallmark gene set enrichment analysis for hepatotoxicity assessment.

## Materials and methods

### Chemical selection

Fourteen chemicals representing different drug classes were selected from Open TG-GATEs for HTTr by TempO-Seq assays (Table 1). Chemicals were tested at low, middle and high concentrations in Open TG-GATEs, and the ratio between the concentrations was 1:5:25 (Igarashi et al., 2014). The low and high concentrations were included in this study. All of the selected chemicals are pharmaceuticals except for WY14643, which is a peroxisome proliferator-activated receptors (PPARs) agonist that has not been approved for clinical use (Pollinger and Merk, 2017). According to DILIrank, which is a data set that ranks the risk of drugs for causing drug-induced liver injury (DILI) in humans, the selected chemicals include drugs which are classified as most-DILI-concern (acetaminophen, diclofenac, indomethacin, isoniazid, ketoconazole, sulfasalazine and valproic acid), less-DILI-concern (doxorubicin, naproxen, ranitidine and simvastatin), and no-DILI-concern (caffeine and chloramphenicol) (Chen et al., 2016). Importantly, several chemicals were reported in the literature for causing stress or injury in rat liver or hepatocytes, indicating hepatotoxic effects in rodents as well (see references in Table 1). Thus, the selected chemicals were expected to induce diverse transcriptomic responses in primary rat hepatocytes. Indeed, analysis of the Open TG-GATES microarray data demonstrated that the chemicals and associated concentrations differentially perturbed the transcriptome of cultured rat hepatocytes, with the number of DEGs (adjusted *p* < 0.05) ranging from zero (indomethacin and chloramphenicol at the respective low concentrations) to more than sixteen thousand (valproic acid at high concentration) after 24 h of treatment.

**Table 1:** Chemicals and associated concentrations tested in this study. References to chemical-induced liver injury in rodents, and the number of differentially expressed genes (adjusted *p* < 0.05) induced in primary rat hepatocytes after 24 h treatment (n = 3) were shown.

Chemical	Drug Class	DILI concern	References (PMID)	Conc. (μM)	ST		WT	
					Batch 1	Batch 2	Batch 1	Batch 2
Acetaminophen	NSAIDs	Most	22,980,195	400	16	189	103	250
				10,000	1228	1360	6838	8002
Diclofenac	NSAIDs	Most	11,870,366	16	5	45	10	113
				400	1031	1244	5163	6497
Indomethacin	NSAIDs	Most	30,252,991	12	4	68	17	218
				300	447	710	966	3462
Isoniazid	Anti-tuberculosis agents	Most	9,029,273	400	11	59	13	108
				10,000	1167	1354	5822	7272
Ketoconazole	Anti-fungal agents	Most	–	0.6	1	355	0	1070
				15	226	589	673	2166
Sulfasalazine	Anti-infective agents	Most	27,340,618	4	68	228	226	667
				100	775	1002	4112	4972
Valproic acid	Anti-convulsants	Most	15,858,223	400	2	8	2	12
				10,000	585	769	2087	3177
Doxorubicin	Anti-neoplastic agents	Less	23,612,702	0.08	16	100	72	424
				2	706	1044	3307	4948
Naproxen	NSAIDs	Less	30,595,947	80	286	273	1028	950
				2000	792	1043	3800	5115
Ranitidine	Anti-ulcer agents	Less	–	160	12	17	15	20
				4000	986	1217	5461	6218
Simvastatin	Anti-lipemic agents	Less	23,761,184	2.4	125	177	231	311
				60	458	479	1493	1793
Caffeine	CNS stimulants	No	–	400	49	240	299	1307
				10,000	471	676	1945	4787
Chloramphenicol	Anti-infective agents	No	–	18	433	740	1549	2668
				450	929	1232	5045	6796
WY14643	PPARα agonists	–	–	8	732	981	3702	5661
				200	777	941	3670	4664

### Hepatocytes isolation

Hepatocytes were harvested from male Wistar rats (250–300 g, InVivos Pte Ltd, Singapore) based on a modified *in situ* collagenase perfusion method described previously (Seglen, 1976). Animals were handled according to the IACUC protocols approved by the IACUC committee of National University of Singapore. The yield of hepatocytes yields was more than 2 × 10^8^ per rat with greater than 90% viability as determined by trypan blue exclusion assay. Isolated hepatocytes were maintained in William’s medium E supplemented with 1 mg/mL BSA, 0.5 µg/mL insulin, 2 mM L-glutamine, 100 nM dexamethasone, 10 ng/mL EGF, 100 μg/mL streptomycin and 100 U/mL penicillin.

### Hepatocyte culture and chemical treatments in collagen sandwich

To coat collagen on a 96-well plate (Thermo Scientific™ Nunc™), we diluted bovine type I collagen (PureCol®, 3 mg/mL, Advanced BioMatrix) with equal volume of 0.01 M HCl and dispensed 80 µL of the diluted collagen solution to each well. After 3 h of incubation at 37 °C, the collagen solution was removed and the wells were washed with 100 µL of PBS twice. Collagen-coated plates were either immediately seeded with cells or stored at 4 °C in PBS for at most 3 days. On the day of cell seeding, 50 µL of culture medium was added to the wells followed by 50 μL of hepatocyte suspension (5 × 10^5^ cells/mL), the latter was placed in a reagent reservoir and transferred using a multi-channel pipette fitted with wide-bore tips. The reagent reservoir was gently rocked between each withdrawal to prevent hepatocytes from settling. After cell seeding, the plate was left undisturbed for 5 min before transferring to a cell culture incubator at 37 °C and 5% CO_2_. The cells were allowed to attach to the collagen-coated surface for 3 h, then the medium was removed and the wells were washed with PBS once to remove any unattached cells or cell debris. Next, 100 μL of collagen solution (0.1 mg/mL in culture medium) was added for forming the top collagen layer. The collagen solution was prepared by first neutralizing 280 µL of stock collagen (3 mg/mL) with 210 µL 1X PBS, 35 µL 10X PBS and 35 µL 0.1 M NaOH, then 0.5 mL of the neutralized collagen was mixed with 7 mL of chilled culture medium. After overnight incubation (16 h), a collagen overlay was formed and the spent medium was replaced with 100 µL of fresh culture medium. Cells were incubated for an additional day before chemical treatment. Care was taken during the removal and addition of culture medium to avoid disturbing the collagen overlay. On the day of treatment, the spent medium was replaced with 100 µL of fresh medium containing chemicals. All treatments were performed in culture medium containing 0.5 % DMSO for 24 h. Each treatment condition was tested in triplicates to examine technical reproducibility. To avoid edge effects due to different rate of evaporation, only the inner 60 wells were used while the remaining 36 perimeter wells were filled with 200 µL of RNAse-free water. Two identical sets of chemically-treated hepatocytes were prepared, one for cell viability assay and the other for TempO-Seq assay. The entire experiment was performed twice using hepatocytes isolated from different individuals in order to examine reproducibility between biological replicates.

### Cell viability assessment

Cell viability was determined using the CellTiter-Glo® luminescent assay, which quantifies the amount of ATP from metabolically active cells. After 24 h of chemical treatment, the spent medium was removed and 200 µL of assay reagent containing equal volume of CellTiter-Glo reagent and cell culture medium was added to the wells. After incubating the plate at r.t. for 10 min to stabilize the luminescence signal, 180 µL of the assay reagent was transferred to a black 96-well plate and luminescence was read using a Tecan M500 microplate reader. Cell viability was expressed as percentage of luminescence intensity of treatment group relative to vehicle control (0.5 % DMSO).

### TempO-Seq transcriptomics assay

To prepare samples for TempO-Seq, we removed the spent medium after 24 h of chemical treatment and washed the wells with 100 µL of PBS twice. Then the cells were lysed by adding 20 µL of 1X TempO-Seq lysis buffer (BioSpyder) in PBS. The plates were incubated at r.t. for 10 min and sealed with Nunc™ Sealing Tapes prior to storage at −80 °C until shipment. Samples were shipped frozen on dry ice to BioSpyder Technologies, Inc. (Carlsbad, CA, USA), where libraries and targeted sequencing of the S1500+ surrogate gene set (ST: 2654 probes; version 1.2) and the whole transcriptome (WT: 22,253 probes; version 1.0) were performed. Briefly, 2 μL of each sample lysate was hybridized with detector oligos from the TempO-Seq rat ST or WT assay, with 1 h hybridization period for the ST assay and overnight for the WT assay. After ligation and nuclease digestion, the ligated products were added into an amplification mix with sample-specific PCR primer pairs, which include the standard Illumina adaptor and a sample-specific barcode sequence. Amplicons were pooled and purified using a PCR clean-up kit (Macherey-Nagel, Mountain View, CA, United States). Sequencing was performed on an Illumina HiSeq 2500 system with both ST and WT libraries included in the same sequencing run. Reads were de-multiplexed using Illumina’s bcl2fastq software version 2.20 into FASTQ files for each sample. Reads were aligned to reference genome (rn6) using STAR aligner version 2.5.3a allowing 2 mismatches. The Tempo-SeqR package (BioSpyder Technologies, Inc., version 1.0) was used to generate read counts data. FASTQ files and read counts data have been deposited in the National Center for Biotechnology Information Gene Expression Omnibus (GEO) (GSE152128).

### Analysis of read counts

All probes in the TempO-Seq assays were included for calculating the total number of read counts, the percentage of probes with zero read count, and the correlation of read counts between technical or biological replicates. Normalization and pre-filtering were not performed for these calculations and the raw read counts data were used. For correlation of read counts, a Pearson correlation coefficient was calculated for each replicate in comparison to the other two replicates form the same batch of samples (between technical replicate 1 and 2, 1 and 3, and 2 and 3), resulting in three correlation coefficients for each treatment. In addition, Pearson correlation coefficients were calculated between replicates from different batches of samples (between replicate 1 of batch 1 and 2, replicate 2 of batch 1 and 2, and replicate 3 of batch 1 and 2) to give the correlation coefficients between biological replicates. For visualization of batch effect by PCA plot, probes with less than an average of ten counts across samples from both batches were excluded from analysis and read counts were normalized by DESeq and varianceStabilizingTransformation (vst) functions of the DESeq2 package (Love et al., 2014).

### Differential gene expression analysis

Probes with less than an average of ten counts across samples from both batches were removed from the count matrix. The resulting count matrix was used as input in the DESeq2 R package to identify differentially expressed genes relative to vehicle control. Probes with adjusted *p* value of<0.05 were considered as differentially expressed. For comparison of DEGs between biological replicates, differential gene expression analysis was independently performed for each batch of samples. For comparisons of DEGs between ST and WT assays and gene set enrichment analysis (GSEA), read counts of gene isoforms were combined by addition to produce one read count per gene, and then differential gene expression analysis was performed with batch correction.

### Gene set enrichment analysis (GSEA)

GSEA was performed using the fgsea R package (Sergushichev, 2016) and the hallmark (h.all.v7.0.symbols.gmt) from MSigDB (Subramanian et al., 2005). Gene symbols and corresponding statistics from DESeq2 analysis were used as input for fgsea analysis with 1000 permutations.

### Connectivity mapping

The inter-platform reproducibility of the TempO-Seq HTTr profiles was evaluated by comparison with the Open TG-GATEs database (Igarashi et al., 2014). using connectivity mapping (Lamb, 2007, Musa et al., 2017). The Open TG-GATEs rat hepatocyte data set was downloaded from ArrayExpress (Athar et al., 2019) under accession number E-MTAB-797 (available from https://www.ebi.ac.uk/arrayexpress/experiments/E-MTAB-797/). Raw CEL files for each treatment, generated using the Affymetrix GeneChip Rat Genome 230 2.0, were normalized using robust multiarray average (RMA) (Irizarry et al., 2003). The difference between the log2 normalized intensity values for 18,894 transcripts for each treatment were compared with matched controls to calculate the average log2 fold-change (L2FC) profiles across 12,962 genes. Probes in each L2FC profile were mapped to genes using the Affymetrix Rat Genome 230 2.0 Array annotation obtained from Ensembl Release 102 (Yates et al., 2020). This resulted in a reference transcriptomic database containing 1,177 profiles associated with 131 chemical treatments at 3 concentrations and 3 time points (2, 8 and 24 h). The L2FC profiles for the TempO-Seq arrays (ST and WT) were used to generate the gene set “signatures” of the 100 to 1000 most up- and down-regulated genes (using a step size of 100). The signatures for each treatment were compared with profiles in the reference database using five different connectivity scoring algorithms including: gene set total enrichment score (gtes) (Iorio et al., 2009, Mootha et al., 2003, Subramanian et al., 2005), extreme cosine (xc) score (Cheng et al., 2014), Spearman correlation coefficient (xcs), Pearson correlation coefficient (xcp) and signed Jaccard index (sji) (Z. Wang et al., 2016). In addition, we also evaluated the impact of restricting the reference profile length to the 1000 to 6000 (which is close to the entire set of genes in the array) most up- and down-regulated genes using a step size of 1000. For each of the 14 chemicals, the rank of the correct hit (based on chemical identity) was identified by connectivity mapping between the signatures from the TempO-Seq data and profiles from the Open TG-GATEs data. The ranks for all chemicals were aggregated across ST and WT data, signature length, profile length and connectivity scoring algorithms to calculate the fraction of chemicals for which the correct hit was also the highest scoring match (Fr1) and the fraction of chemicals for which the correct hit was in the top 5 and 10 highest scoring matches (Fr5 and Fr10, respectively).

## Results

In this study, primary rat hepatocytes were maintained in collagen sandwich in 96-well plates and treated for 24 h with a panel of 14 chemicals at two different concentrations, totaling twenty-eight treatments (Table 1). The chemicals, associated concentrations and treatment duration were selected from Open TG-GATEs, in which the highest concentration for soluble compounds was defined as the concentration yielding an 80–90% relative survival ratio through DNA quantification, whereas for poorly soluble compounds the highest concentration was defined as the maximum solubility in vehicle (up to 0.5% DMSO) (Igarashi et al., 2014). Herein, the viability of rat hepatocytes after 24 h of exposure to chemicals was measured by ATP quantification, which is an indicator of metabolically active cells. Viability was found to be greater than 80% for all treatments except indomethacin (78.1%) and valproic acid (74.3%) at the respective high concentrations (Supplementary Table 1). Thus, the majority of treatments were non-toxic to primary rat hepatocytes and unspecific gene signatures associated with cell death could be avoided.

### Evaluating technical and biological reproducibility of the HTTr platform

Establishing the reproducibility of a high-throughput platform is a prerequisite in incorporating the acquired data for regulatory risk assessment; hence the first objective of this study is to evaluate the reproducibility of TempO-Seq® data of primary rat hepatocytes in collagen sandwich culture. To examine the reproducibility between technical replicates, each chemical treatment was performed in triplicate in three adjacent wells on the same multi-well plate. To examine biological, or batch-to-batch, reproducibility, the entire experiment was repeated using rat hepatocytes isolated from another individual with library construction and sequencing independently performed from the first batch of samples.

**Fig. 1:**
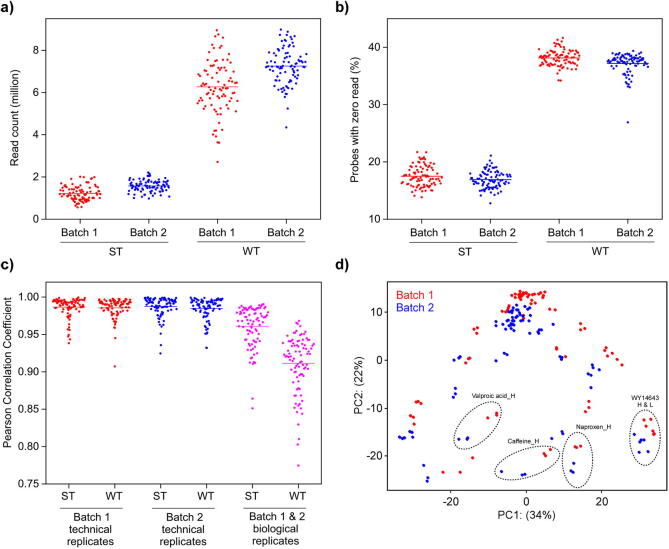
Summary of read count data. Each dot represents (a) the read count of a sample in millions, (b) the percentage of probes with zero read count, and (c) Pearson correlation coefficient of read counts between technical replicates within the same experiment or between biological replicates from two independent experiments. Lines indicate the mean values. (d) PCA plot of variance stabilized counts of samples ran by ST assay; each dot represents one sample. The high and low doses of each chemical are signified by H and L, respectively.

We first analyzed the read count data to establish a general trend of platform performance. The read depth (millions) of samples ran by ST assay ranged from 0.6 to 2.0 (mean = 1.2 ± 0.3) and from 1.0 to 2.2 (mean = 1.6 ± 0.3) for batch 1 and 2, respectively (Fig. 1a). For samples ran by WT assay, the read depth ranged from 2.7 to 9.0 (mean = 6.2 ± 1.2) and from 4.4 to 9.0 (mean = 7.3 ± 0.9) for batch 1 and 2, respectively (Fig. 1a). Compared with samples in batch 1, the overall read depth was higher for samples in batch 2. Compared with WT assay, the read depth is approximately 5X lower for ST assay as it targets a smaller number of genes. It was observed that a significant fraction of probes has zero read count (Fig. 1b). In batch 1, the fraction of probes with zero read count averaged 17.5 ± 1.8% per sample for ST assay, while up to 38.1 ± 1.4% of probes has zero read count for WT assay. A marginal decrease in the fraction of probes with zero read count was observed for samples in batch 2 compared to batch 1, which was attributed in part to the higher read depth that increased the sensitivity of detecting lowly expressed genes. Further analysis showed that the fractions of probes which consistently have zero read count across all samples from both batches were 0.04 and 1.9% for ST and WT assay, respectively.

Pearson correlation of read counts was performed to assess reproducibility between technical replicates within the same batch of samples and between biological replicates from different batches of samples. Regardless of the batch or the type of assay, correlation coefficients of read counts between technical replicates were above 0.90 for the various treatments (Fig. 1c). Correlation coefficients between biological replicates were generally lower than between technical replicates with values ranging from 0.85 to 0.99 for ST assay and 0.77 to 0.97 for WT assay (Fig. 1c). For correlation between read counts of different chemicals, stronger correlations were typically observed between low concentration samples than high concentration samples, which presumably were due to low concentration treatments inducing weak transcriptomic responses that resembled each other (Supplementary Figs. 1 and 2). PCA plot of variance-stabilized counts illustrated batch-to-batch variations as evident by the separation of biological replicates from the two batches of samples (Fig. 1d). Overall, the results indicated that the HTTr platform exhibited high reproducibility between technical replicates and variability in cell source and/or sequencing run contributed to the decreased reproducibility between biological replicates.

Next, differential gene expression analysis was independently performed for the two batches of samples. A number of observations were made by comparing the number of DEGs between different assays, batches and treatments (Table 1). First, it was observed that the number of DEGs varied between treatments with 0.6 µM ketoconazole of batch 1 inducing the least number of DEGs (1 and 0 for ST and WT assay, respectively) and 10 mM acetaminophen of batch 2 inducing the greatest number of DEGs (1360 and 8002 for ST and WT assay, respectively). Second, the number of DEGs detected by ST assay was lower than WT assay, which was expected as the ST assay targets a smaller number of genes. Third, for the same chemical treatment, batch 2 generally had more DEGs compared to batch 1. Lastly, high drug concentrations typically resulted in more DEGs compared to low drug concentrations, except for WY14643 that had a similar number of DEGs at both concentrations.

**Fig. 2:**
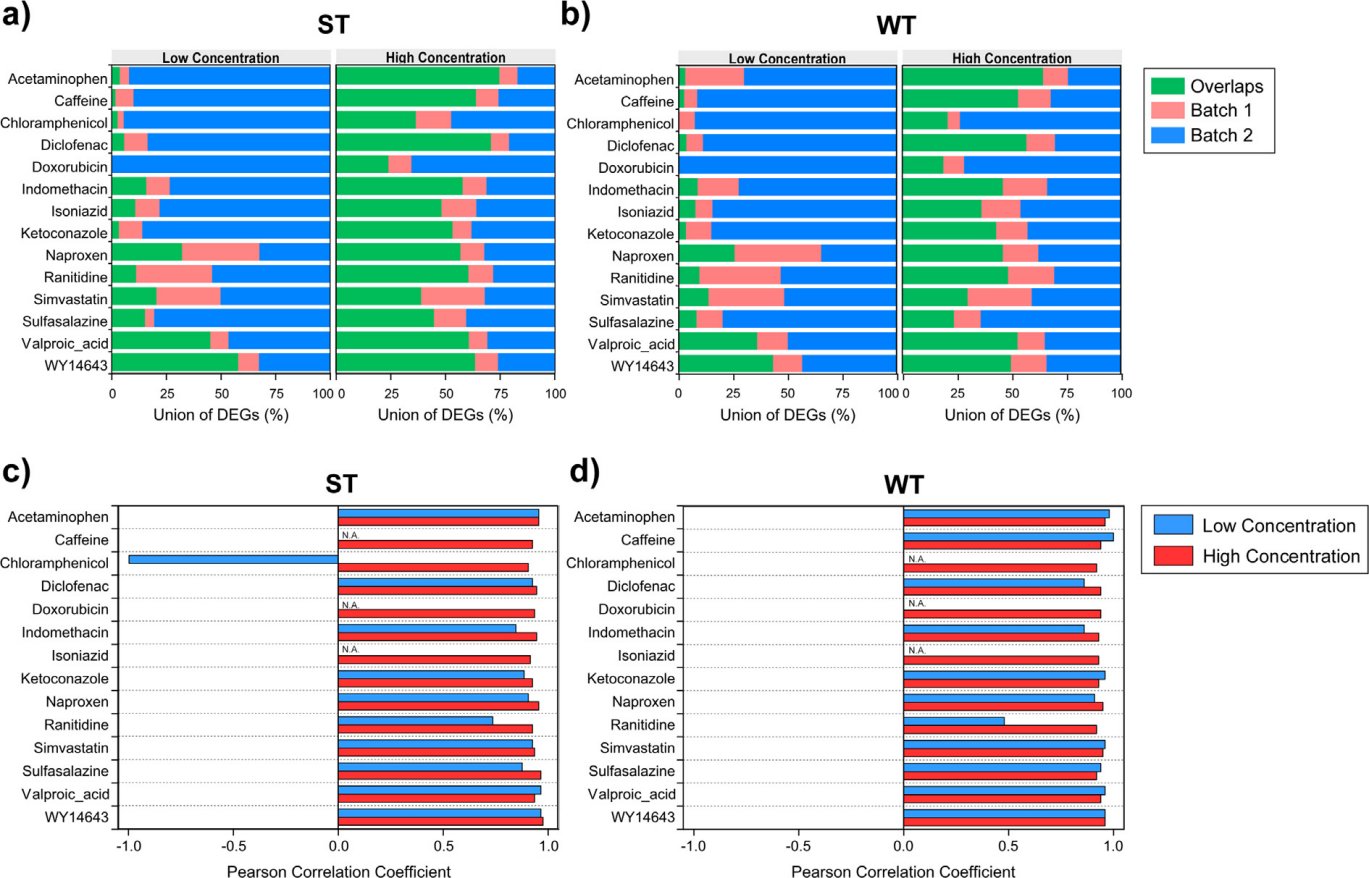
Reproducibility of differential gene expression between biological replicates. Fraction of DEGs (adjusted *p* < 0.05) identified in both batches (overlaps) or exclusively in batch 1 or 2 for (a) ST and (b) WT. Pearson correlation coefficients of L2FC of overlapping DEGs between biological replicates for (c) ST and (d) WT.

Reproducibility of differential gene expression was evaluated by determining the fraction and correlation of DEGs that overlapped between the two batches of samples (biological replicates). It was found that treatment with high concentrations of drugs typically produced a more reproducible transcriptomic signature than treatment with low concentration of drugs. For example, the fraction of overlapping DEGs between the two batches of acetaminophen-treated samples ran by ST assay increased from 4% at 400 µM to 75% at 10 mM (Fig. 2a). The fraction of overlapping DEGs with increasing treatment concentration was also observed for samples ran by WT assay (Fig. 2b). Pearson correlation coefficients of L2FC values of overlapping DEGs were above 0.9 for high concentration treatments in both ST (Fig. 2c) and WT assay (Fig. 2d). In contrast, some low concentration treatments had no overlapping DEGs, others had a lower correlation coefficient compared to the high concentration counterpart, and one treatment had a negative correlation coefficient (18 µM chloramphenicol ran by ST assay) (Fig. 2c, d). It is worth mentioning that some genes were differentially expressed in the opposite direction as treatment concentration increased from low to high (Supplementary Fig. 3), which underscores the importance of testing multiple concentrations in order to establish dose-dependent transcriptomic signatures. Besides concentration, reproducibility of differential gene expression also depended on the potency of a drug in perturbing the transcriptome of rat hepatocytes. For instance, at 400 µM, diclofenac induced 5X more DEGs than caffeine as detected by ST assay (Table 1, batch 2), and had 71% of overlapping DEGs between biological replicates compared to 2% of caffeine (Fig. 2a, b).

### Evaluating inter-platform reproducibility by connectivity mapping

The second objective of this work is to evaluate inter-platform transcriptomic reproducibility by comparing TempO-Seq ST and WT data generated in this study with Affymetrix rat GeneChip 230 2.0 data produced by the Open TG-GATEs project (Igarashi et al., 2014). The gene signatures for each of the chemical treatments from the ST and WT profiles were searched against the 1177 Open TG-GATES profiles using five different connectivity-scoring algorithms. As an illustrative example, connectivity mapping results for the 200 most up- and 200 down- regulated (nq = 200) ST gene signature for the 400 µM valproic acid treatment with the 1000 up- and down-regulated genes (nr = 1000) in the reference profiles are shown in Table 2. Using the sji algorithm for connectivity-scoring, the highest-scoring match for 400 µM valproic acid was the identical treatment of 400 µM valproic acid treatment for 24 h (valproic-acid-400.00uM-24 h-rn-hep-e-mtab-797). The second-highest scoring match was also with valproic acid but for a treatment concentration of 2000 µM for 24 h (valproic-acid-2000.00uM-24 h-rn-hep-e-mtab-797). Using gtes for connectivity-scoring also produced the top two “hits” with valproic acid; however, the reference profile for the 2000 µM 24 h valproic acid treatment produced a greater score than the 400 µM 24 h valproic acid treatment. The best hit using xc algorithm was for the 300 µM 24 h clofibrate treatment (clofibrate-300.00uM-24 h-rn-hep-e-mtab-797) and the 400 µM 24 h valproic acid treatment was the second hit. The correct chemical was identified as the hit for sji, gtes and xcp, and as top five hits for xc and xcs. Based on our connectivity mapping analysis, the valproic acid gene signature (nq = 100) was highly reproduced between the TempO-Seq ST and Open TG-GATES platforms. We systematically analyzed the connections between the ST and WT profiles for all treatments using different signature lengths, profile lengths, and connectivity-mapping algorithms, and our results are provided as supplemental material (Supplementary Table 2).

**Table 2:** Connectivity between TempO-Seq ST gene signature (400 µM of valproic acid) and the TG-GATEs Affymetrix profiles. Connectivity analysis was performed by querying the top 200 up- and down-regulated genes of the TempO-Seq signature against the top 1000 up- and down-regulated genes in the reference profile using five different connectivity-mapping algorithms including: signed Jaccard index (sji), gene set total enrichment score (gtes), extreme cosine (xc) score, Pearson correlation coefficient (xcp) and Spearman correlation coefficient (xcs). The top 10 hits are ranked by the sji score as shown below. The Open TG-GATES treatment identifiers were formed by concatenating the chemical, treatment concentration (µM), treatment duration (24 h), species (rat), cell type (hepatocyte) and the Array Express record identifier for the study (E-MTAB-797).

Rank	Open TG-GATES Treatment	sji	gtes	xc	xcp	xcs
1	valproic-acid-400.00uM-24 h-rn-hep-e-mtab-797	0.413	3.435	0.857	0.817	0.772
2	clofibrate-300.00uM-24 h-rn-hep-e-mtab-797	0.352	3.371	0.86	0.805	0.791
3	wy-14643–8.00uM-24 h-rn-hep-e-mtab-797	0.317	3.274	0.842	0.795	0.779
4	wy-14643–40.00uM-24 h-rn-hep-e-mtab-797	0.296	3.269	0.836	0.788	0.76
5	valproic-acid-2000.00uM-24 h-rn-hep-e-mtab-797	0.398	3.638	0.813	0.786	0.723
6	simvastatin-60.00uM-24 h-rn-hep-e-mtab-797	0.349	1.99	0.805	0.768	0.708
7	clofibrate-60.00uM-24 h-rn-hep-e-mtab-797	0.268	2.046	0.824	0.752	0.785
8	fenofibrate-30.00uM-24 h-rn-hep-e-mtab-797	0.233	1.909	0.806	0.749	0.723
9	wy-14643–200.00uM-24 h-rn-hep-e-mtab-797	0.255	3.313	0.765	0.729	0.63
10	tolbutamide-2000.00uM-24 h-rn-hep-e-mtab-797	0.277	2.123	0.649	0.649	0.647

**Fig. 3:**
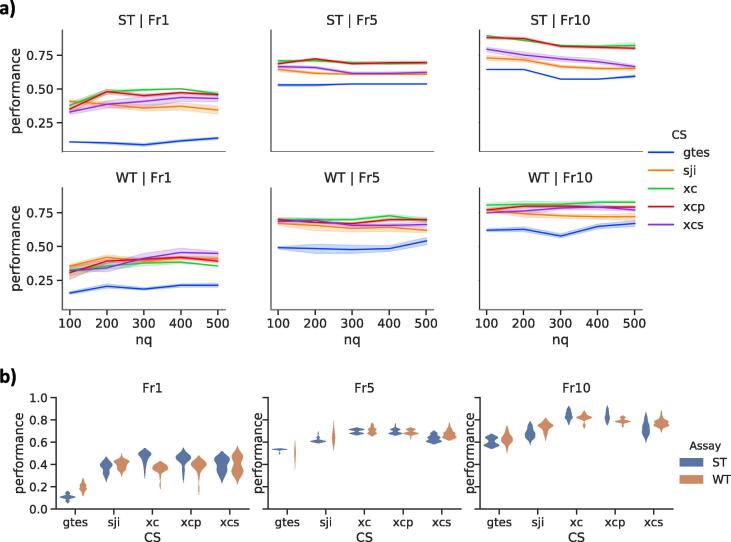
The performance of connectivity mapping using the surrogate transcriptome (ST) and the whole transcriptome (WT) signatures. Performance was evaluated by the fraction of chemical treatments correctly identified as the best match (Fr1), in the top 5 matches (Fr5) or in the top 10 matches (Fr10). Connectivity-scoring (CS) algorithms include: signed Jaccard index (sji), gene set total enrichment score (gtes), extreme cosine (xc) score, Pearson correlation coefficient (xcp) and Spearman correlation coefficient (xcs). (a) Performance comparisons for different signature lengths (nq) across all reference profile lengths (nr). (b) Performance distributions for different CS comparing ST and WT across all nq and nr.

The performance results for connectivity mapping using ST and WT signatures of different lengths and different algorithms are shown in Fig. 3a. Across all analysis choices, the mean Fr1 was 0.28 (SD = 0.17) for ST and 0.29 (SD = 0.12) for WT, the mean Fr5 was 0.58 (SD = 0.12) for ST and 0.58 (SD = 0.15) for WT, and mean Fr10 was 0.68 (SD = 0.14) for ST and 0.68 (SD = 0.16) for WT. The differences in performance between connectivity-scoring algorithms for the Fr1, Fr5 and Fr10 metrics using ST and WT across all signature and profile lengths are shown in Fig. 3b (and complete results are provided as Supplementary Table 3). The highest Fr1 performance for ST signatures was observed for xc (mean = 0.46 and SD = 0.05) while sji and xcs were tied for the WT signatures (mean = 0.40 and SD = 0.04, and mean = 0.40 and SD = 0.07, respectively). The highest Fr5 performance for ST was observed for xc and xcp (mean = 0.7 and SD = 0.02) while xc produced the highest performing WT signatures (mean = 0.70 and SD = 0.03). Finally, the highest Fr10 performance for ST and WT was produced by xc (mean = 0.84 and SD = 0.03, and mean = 0.82 and SD = 0.03, respectively). When compared by individual connectivity-scoring algorithms, we found the ST signatures were slightly (1.2-fold) more accurate than WT signatures and xc generally performed better that other algorithms.

There was a complex relationship between the length of signatures, connectivity-scoring algorithms and transcriptome assays (ST vs WT), but we observed some interesting trends. Increasing the signature length did not result in substantial improvement in performance for any of connectivity-score algorithms. The Fr1 performance of ST signatures for xc increased up to nq ≤ 300 and then decreased whereas the performance for sji decreased monotonically for nq > 100. On the other hand, increasing the WT signature length produces modest improvements in performance for sji (nq ≤ 200), xcs (nq ≤ 400) and gtes (nq ≤ 400). The Fr5 performance of ST signatures varied less with nq as it did for Fr1. For the WT signatures, Fr5 signatures showed consistent performance for all algorithms except gtes. Finally, the Fr10 performance of ST signatures generally decreased for nq ≥ 200. On the other hand, the Fr10 performance of WT signatures did not change much for xc, xcp and xcs with nq, it decreased for sji and it increased for gtes.

The best connectivity mapping approaches for the ST and WT signatures were identified for the Fr1, Fr5 and Fr10 performance metrics. The ST signatures produced the highest Fr1 performance of 0.5 using xc (with shortest nq = 200 and shortest nr = 2000) and xcp (with shortest nq = 200 and nr = 4000). The highest Fr1 performance of 0.46 for WT signatures was produced by xcs (with shortest nq = 300 and shortest nr = 4000). The highest Fr5 performance value for ST signatures of 0.75 was produced by xcp (nq = 200 and nr = 5000) and for WT signatures as value of 0.75 was produced by sji, xcs and xc (for different signature and profile lengths). Lastly, the highest Fr10 performance value for ST signatures of 0.89 was produced by xcp and xc (nq = 100 and nr = 5000) and for WT signatures as value of 0.86 was produced by xcs and xc (for nq = 400 and nr = 5000).

In Table 3, we selected the optimal values of nq and nr for each connectivity-scoring algorithm to report the best rank for the ST and WT signatures. The median and average ranks across all methods were 1 and 5, respectively for the ST signatures, and 1 and 5.5 for the WT signatures. We did not find any hits for the acetaminophen 400 µM, doxorubicin 0.08 µM, ketoconazole 0.6 µM, diclofenac 16 µM and ranitidine 160 µM treatments in the Open TG-GATES database using either ST or WT signatures with any of the five connectivity-scoring algorithms. For the remaining treatments, the best hit was the same chemical for 68% of the treatments, and within the top 10 hits for 95% of the treatments for both ST and WT signatures. The acetaminophen 10000 µM treatment produced the lowest ranking hits across all treatments with minimum ranks of 48 (ST) and 48 (WT). This was followed by the indomethacin 12 µM treatment with a minimum rank of 19 (ST) and 17 (WT), the naproxen 80 µM treatment with a minimum rank of 10 (ST) and 4 (WT).

**Table 3:** Connectivity mapping analysis of best hits. Best ranks for the TempO-Seq surrogate transcriptome (ST assay) and whole transcriptome (WT assay) gene signatures (length nq), in the TG-GATES reference profiles (length nr) for each connectivity-scoring algorithm (CS). Connectivity-scoring (CS) algorithms include: signed Jaccard index (sji), gene set total enrichment score (gtes), extreme cosine (xc) score, Pearson correlation coefficient (xcp) and Spearman correlation coefficient (xcs). The last two rows show the median and mean ranks by each CS method and the last two columns show the best (minimum) rank identified for each treatment in each assay.

	Assay	ST					WT						
	CS	gtes	sji	xc	xcp	xcs	gtes	sji	xc	xcp	xcs	Min. Rank	
	nq	400	100	200	200	300	200	200	200	200	300		
	nr	3000	2000	3000	2000	4000	2000	2000	4000	4000	4000	ST	WT
Chemical	Conc (µM)												
Acetaminophen	1000	119	48	55	56	53	65	61	58	60	62	48	58
Caffeine	400	4	1	2	1	1	1	1	2	1	1	1	1
	10,000	1	1	1	1	1	1	1	1	1	1	1	1
Chloramphenicol	18	15	22	4	9	10	120	7	6	18	19	4	6
	450	1	1	1	1	1	18	1	1	1	1	1	1
Diclofenac	400	5	1	1	1	1	8	1	1	1	1	1	1
Doxorubicin	2	10	1	1	1	1	3	1	3	3	1	1	1
Indomethacin	12	19	20	26	19	28	17	30	18	19	29	19	17
	300	22	1	1	2	1	23	1	1	1	1	1	1
Isoniazid	400	50	6	1	1	7	1	14	6	7	10	1	1
	10,000	35	2	9	10	18	37	1	1	1	1	2	1
Ketoconazole	15	1	2	1	1	1	1	1	1	1	1	1	1
Naproxen	80	24	10	13	10	16	12	6	5	4	4	10	4
	2000	20	1	1	1	1	1	1	1	1	1	1	1
Ranitidine	4000	26	9	1	1	7	10	10	7	9	9	1	7
Simvastatin	2	3	22	6	5	17	1	16	2	2	10	3	1
	60	1	1	1	1	1	1	1	1	1	1	1	1
Sulfasalazine	4	15	27	7	7	17	10	10	11	10	12	7	10
	100	24	12	10	8	10	26	8	8	8	8	8	8
Valproic	400	4	1	1	1	1	14	1	1	1	1	1	1
	10,000	4	1	1	1	1	5	1	1	1	1	1	1
WY14643	8	3	1	1	1	1	6	1	1	1	1	1	1
	200	3	1	1	1	1	4	2	2	2	1	1	1
	Median	10	1	1	1	1	8	1	2	1	1	1	1
	Mean	17.8	8.3	6.3	6.1	8.5	16.7	7.7	6	6.7	7.7	5	5.5

The similarity between TempO-Seq ST and TG-GATES transcriptomics profiles was also driven by common mechanisms of action for the chemicals (Iorio et al., 2013). To illustrate this issue, consider valproic acid, which is an anticonvulsant drug used for treating epilepsy and bipolar disorder, however, it is also known to affect the peroxisome proliferator activated receptors (PPARs) in the liver (Szalowska et al., 2014), causing changes in lipid metabolism and resulting in hepatic steatosis (Amacher and Chalasani, 2014). The high-scoring matches for the valproic acid 400 µM treatment (Table 2) in Open TG-GATES included valproic acid as well as simvastatin, clofibrate, tolbutamide, WY-14643, diclofenac and gemfibrozil, which alter lipid metabolism through PPARs (Rakhshandehroo, Hooiveld, Müller, and Kersten, 2009). This is an encouraging result as we are interested in using the rat liver HTTr platform to screen potential hepatotoxicants.

Overall, the connectivity mapping analysis of these 14 chemicals using TempO-Seq ST and WT data and Affymetrix rat GeneChip data from Open TG-GATES on rat primary hepatocytes show a high-level of inter-platform reproducibility.

### Comparison between gene signatures of TempO-Seq ST and WT assays by DEGs

**Fig. 4:**
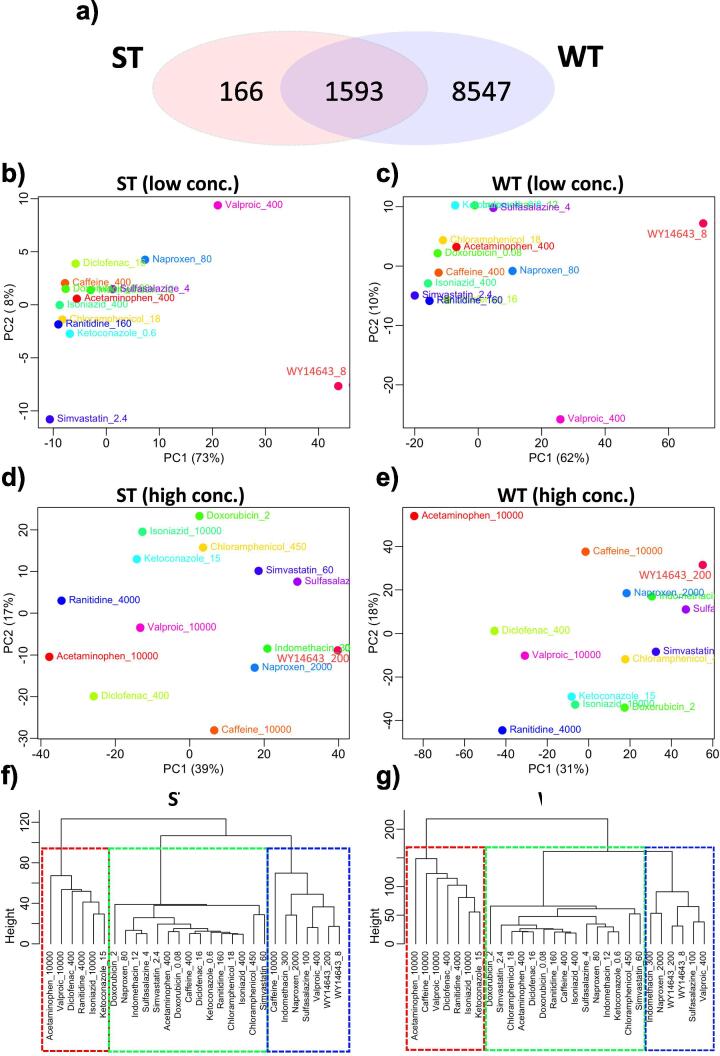
Comparison of gene signatures generated by ST and WT assay. (a) Venn diagram depicting the overlap of the union of DEGs between ST and WT assays. PCA plot using the L2FC values of the union of DEGs for (b and c) low and (d and e) high treatment concentrations. Unsupervised hierarchical clustering (Euclidean distance and ward.D2 algorithm) of samples using L2FC values of DEGs for (f) ST and (g) WT assay.

The third objective of this study is to evaluate the robustness of S1500+ gene set as a surrogate for the whole transcriptome. A positive correlation of L2FC values was observed between genes common to both assays, with correlation coefficients being above 0.95 for all treatments (Supplementary Fig. 4). By generating a union of DEGs across all treatments for each assay, 1593 DEGs were found to overlap between the two assays (Fig. 4a), with additional 166 and 8547 DEGs identified by the ST and WT assay, respectively. Among the 166 DEGs identified by ST assay alone, half were detected using probe sequences that were also found in the WT assay but annotated with different gene symbols; hence they were not identified as overlapping DEGs between the two assays through the matching of gene symbols. Another 25% were genes that were annotated with the same gene symbols as in the WT assay but detected using different probe sequences. The remaining were genes that were found only in the ST assay.

PCA plots were generated using the L2FC values of the union of DEGs to visualize and compare the gene expressions between the two assays (Fig. 4b–e). Remarkable similarities were observed between PCA plots of ST and WT for low concentration treatments. The majority of treatments were situated on the left of the plots except for 400 µM valproic acid and 8 µM WY14643 which were located away from the main group (Fig. 4b and c). High concentration treatments were farther apart from each other on the PCA plots for both ST and WT, suggesting that the chemical-induced transcriptomic responses were more distinct than those at low concentrations (Fig. 4d and e). Unsupervised hierarchical clustering of samples ran by ST assay resulted in three main clusters (Fig. 4f). The left cluster included treatments with acetaminophen, valproic acid, diclofenac, ranitidine, isoniazid, and ketoconazole at their respective high concentrations. It is worth mentioning that the drugs in this cluster belonged to the most-DILI-concern classification. The right cluster included treatments with high concentrations of caffeine, indomethacin, naproxen, and sulfasalazine; low concentration of valproic acid; and low and high concentrations of WY14643. Finally, the middle (intermediate) cluster was characterized by treatments with low concentration of drugs, except chloramphenicol and simvastatin at both low and high concentrations. Unsupervised clustering of samples ran by WT assay also produced three main clusters with the drugs in each cluster being identical to ST assay except for 10 mM caffeine, which was included in the right cluster for ST assay but was found in the left cluster for WT assay (Fig. 4g). The resemblance in PCA plots and hierarchical clustering between ST and WT visually demonstrated that the S1500+ and WT genes capture similar overall transcriptomic effects for these chemical treatments in rat hepatocytes. The observation is consistent with the selection criteria of the S1500+ gene set, which includes maximal capturing of biological diversity in gene expression datasets.

### Comparison between gene signatures of TempO-Seq ST and WT assays by GSEA

Next, gene signatures captured by the ST and WT assays were compared using GSEA with the hallmark gene set collection. The number of genes within each hallmark gene set after restricting the genes present in the ST and WT assay are shown in Supplementary Table 4. By comparing normalized enrichment score (NES) between ST and WT assay, a majority of treatments (26 out of 28) had at least one overlapping hallmark among the top five ranked by descending order of NES between the two assays, with most treatments having three or more (Supplementary Fig. 5a). Moreover, a positive correlation of NES between ST and WT assays was observed with correlation coefficients ranging from 0.5 to 0.9 at low concentration and from 0.7 to 0.9 at high concentration (Supplementary Fig. 5b).

**Fig. 5:**
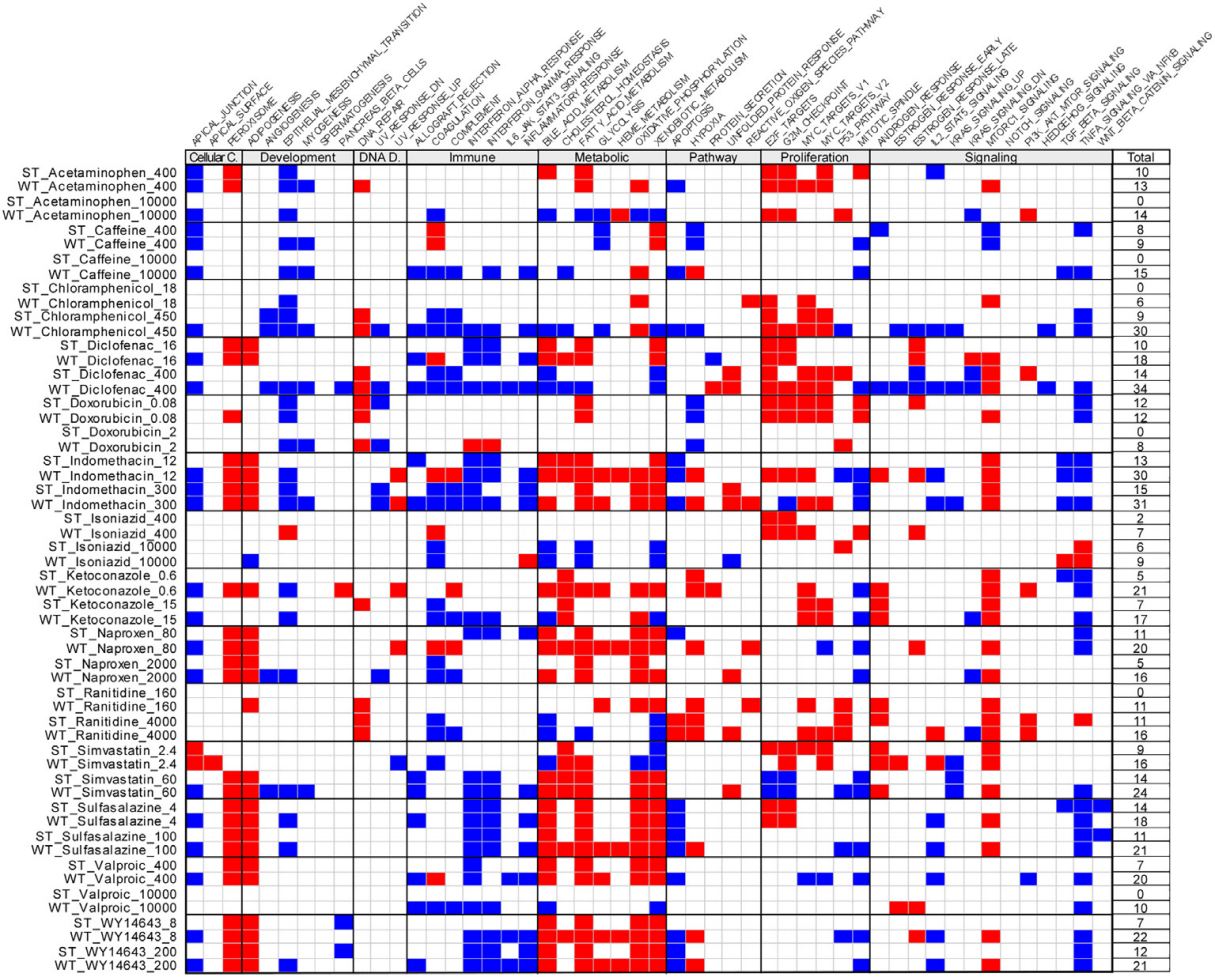
Significantly enriched hallmark gene set (FDR < 0.05) by treatment. Positively and negatively enriched gene sets are indicated by red and blue, respectively. White indicates no statistical significance in the enrichment. The total number of significantly enriched gene sets is listed on the right of the figure. (For interpretation of the references to colour in this figure legend, the reader is referred to the web version of this article.)

**Fig. 6:**
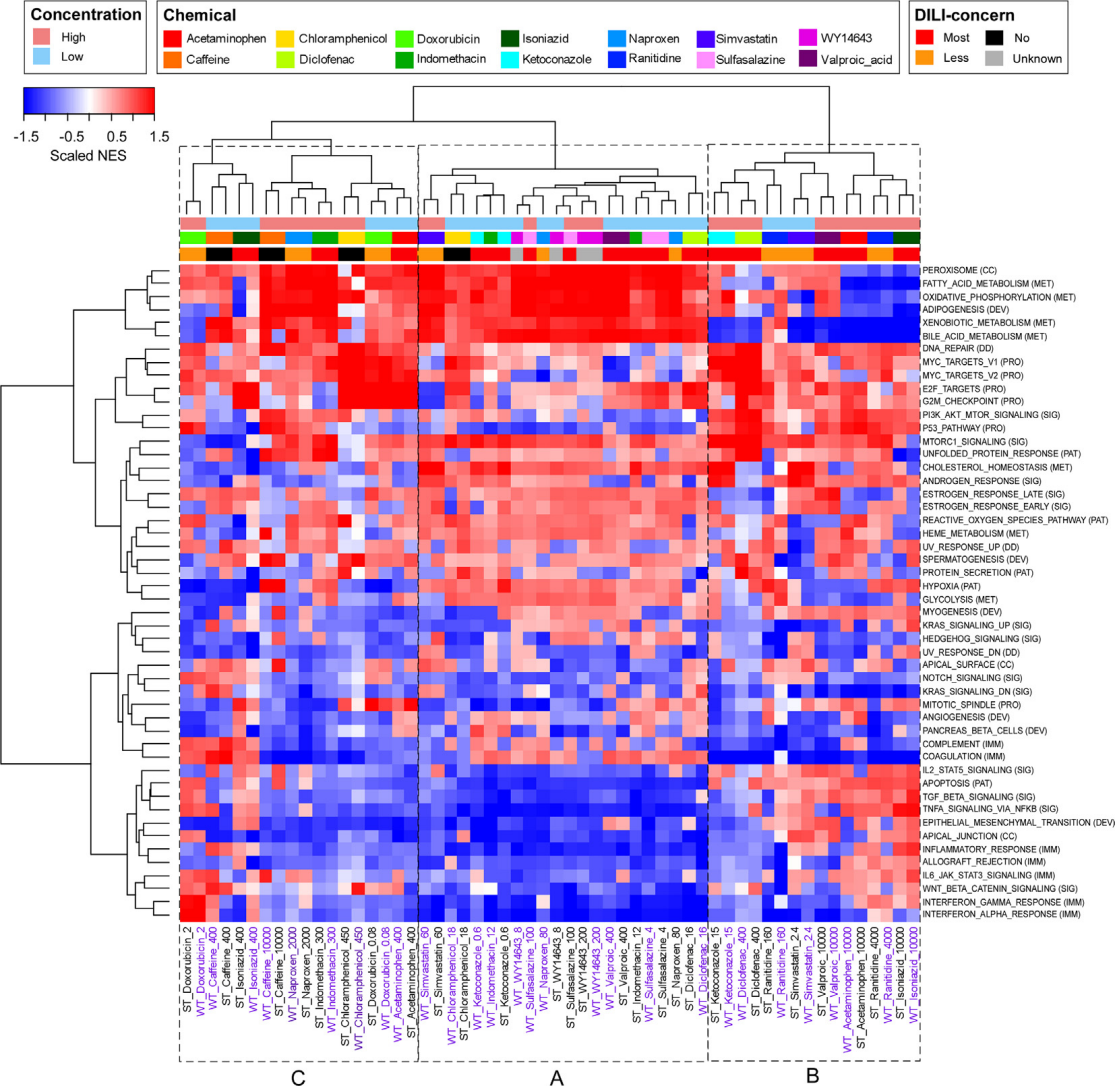
Unsupervised hierarchal clustering using NES of all samples from both batches of experiments using NES (Euclidean distance and ward.D2 algorithm). Column labels of samples ran by ST and WT were in black and purple, respectively. (For interpretation of the references to colour in this figure legend, the reader is referred to the web version of this article.)

It was also observed that ST assay generally resulted in a lower number of significantly enriched hallmark gene sets (FDR < 0.05) compared to WT assay (Fig. 5). Nonetheless, most gene sets significantly enriched in ST assay were also significantly enriched in the WT assay. Notably, the significantly enriched gene sets were consistent with the known activity of the chemicals. As shown in Fig. 5, Fig. 6 hallmark gene sets of the immune category were downregulated by indomethacin in both ST and WT assays, reflecting the anti-inflammatory activity of the NSAID. Sulfasalazine, an anti-infective agent, downregulated both interferon-alpha and gamma response and TNF-alpha signaling via NFκB in both ST and WY assays, which is consistent with its mechanism of action (Chan et al., 2008). WY14643 significantly upregulated peroxisome at both concentrations in both assays, consistent with its mechanism of action as a peroxisome proliferator-activated receptor alpha (PPARα) agonist. In addition, WY14643 downregulated several immune-related hallmarks at high concentration, indicating potential anti-inflammatory activity as reported previously (Briguglio et al., 2010). Moreover, bile acid metabolism, fatty acid metabolism, oxidative phosphorylation and xenobiotic metabolism of the metabolic category were significantly enriched by WY14643, supporting the notion of PPARα as a regulator of hepatic lipid metabolism (Rakhshandehroo et al., 2009). Significant as well as consistent enrichment between ST and WT assays of peroxisome and hallmarks of the metabolic category was observed for valproic acid at low concentration; simvastatin at high concentration; and sulfasalazine, indomethacin, naproxen and simvastatin at both low and high concentrations and, suggesting common transcriptional effects on cultured rat hepatocytes between these drugs and WY14643.

### Differentiation between hepatotoxic and non-hepatotoxic treatments by clustering using NES of hallmark gene set

Hierarchical clustering divided treatments according to similarities in the enrichment of hallmark gene sets. NES of samples calculated by both ST and WT assays were combined into a single matrix for the analysis. The clustering correctly paired samples by chemical treatment, that is, the nearest neighbor to a treatment ran by ST is the same treatment ran by WT (Fig. 6), was observed for all samples except naproxen, indomethacin, ketoconazole and WY14643 at the respective low concentrations, and sulfasalazine at high concentration. This observation further confirmed the above observation that NES scores were highly consistent between ST and WT assays.

Besides producing treatment-specific pairs, hierarchical clustering using NES of hallmark gene set divided the treatments into three major clusters (labeled A-C in Fig. 6). Strongly positive NES characterized cluster A for most hallmarks of the metabolic category, whereas NES for hallmarks of the proliferation category ranged from strongly negative to weakly positive. NES for apoptosis, TNFα via NFκB, inflammatory response, and interferon-alpha response were strongly negative in Cluster A, suggesting that the chemicals and associated concentrations did not induce significant stress on hepatocytes. Cluster B exhibited a contrasting biological state compared to Cluster A with strongly negative to weakly positive NES for hallmarks of the metabolic category and weakly negative to strongly positive NES for hallmarks of the proliferation category. Some treatments in Cluster B showed strongly positive NES for apoptosis, TGFβ signaling, TNFα signaling via NFκB, epithelial mesenchymal transition and inflammatory response, all of which were signaling pathways characteristic of liver injury. Thus, the drugs and associated concentrations in Cluster B caused impairment of metabolic functions and activation of several signaling pathways in response to the stressful chemical treatments. Lastly, Cluster C showed an intermediate biological state between Cluster A and B. For example, NES for hallmarks of metabolic category were predominantly positive as in Cluster A except for cholesterol homeostasis and glycolysis. NES for hallmarks of proliferation category ranged from weakly negative to strongly positive, which more closely resembled Cluster B. Some treatments in Cluster C showed positive NES for apoptosis, TGFβ signaling, TNFα signaling via NFκB, epithelial-mesenchymal transition, and inflammatory response.

Hierarchical clustering of NES of ST samples alone also resulted in three main clusters (Supplementary Fig. 6). The treatments within each cluster were the same as those obtained by clustering NES of both ST and WT samples except for 18 uM chloramphenicol, 16 uM diclofenac and 160 uM ranitidine (compare Fig. 6 to Supplementary Fig. 6). Statistical analysis was performed on NES between Cluster A and Cluster B in Supplementary Fig. 6, which contained the putative non-hepatotoxic and hepatotoxic treatments. Twenty-eight out of the 50 hallmarks showed statistically significant differences (FDR < 0.05) in NES between the two clusters (Supplementary Table 5). The top 5 statistically significant gene sets were xenobiotic metabolism, bile acid metabolism, apoptosis, p53 pathway and coagulation. These five gene sets had an absolute difference in the mean NES between the two clusters of greater than 2.5 and an FDR of less than 5 × 10^-4^.

Hierarchical clustering using NES of the top 5 hallmarks also divided the treatments into three clusters (Supplementary Fig. 7). Twenty-two out of 28 treatments were found in the same clusters as those derived from the entire set of 50 hallmarks (compare Supplementary Fig. 6 to Supplementary Fig. 7). Cluster A (non-hepatotoxic) was characterized by positive enrichment in xenobiotic metabolism, bile acid metabolism, coagulation, and negative enrichment in the p53 pathway and apoptosis, and included only low concentration treatments except for 100 uM sulfasalazine. Cluster B (hepatotoxic) was characterized by impaired coagulation and metabolic functions and apoptosis and p53 pathway activation. The treatments in Cluster B included 5 most-DILI-concern drugs and 3 less-DILI-concern drugs, and all were high concentration treatments except for 2.4 uM simvastatin. Cluster C is an intermediate cluster that exhibited partially impaired hepatic functions (mostly positive enrichment in hepatic metabolism and negative enrichment in coagulation) and a mixed enrichment in p53 and apoptosis. Taken together, the results demonstrated that hepatotoxic treatments could be identified by unsupervised hierarchical clustering using NES of a subset of the hallmark gene set.

## Discussion

There is an urgent need for new high-throughput approaches for testing chemical safety that reduce dependence on whole-animal apical outcomes. *In vitro* tissue/organ models coupled with HTTr are expected to be one of the main approaches in chemical hazard characterization. Various approaches have been developed to analyze and incorporate transcriptomic data for chemical risk assessment. For instance, benchmark dose (BMD) modeling of transcriptomic data has been developed to estimate apical BMD or point-of-departure (POD) values (Gwinn et al., 2020, Johnson et al., 2020, Thomas et al., 2007, Thomas et al., 2013). Transcriptomic signature of a test chemical could also be queried against signatures of reference chemicals through pattern-matching approaches such as connectivity mapping (De Abrew et al., 2016, Lamb et al., 2006) in order to uncover potential modes of action (MOAs) or biological targets of the test chemical for further validation via pathway or target-specific assays. Transcriptomic data has also been recently used to derive predictive gene signatures for early detection of drug-induced liver injury (DILI) (Kang et al., 2020, Monroe et al., 2020, Podtelezhnikov et al., 2020).

In this study, we described an HTTr platform comprised of a collagen sandwich cell culture model and the TempO-Seq transcriptomic assay. Using a comprehensive study design involving fourteen chemicals with different DILI classifications and two treatment concentrations, we systematically evaluated technical (well-to-well), biological (batch-to-batch), and cross-platform (TempO-Seq-to-Affymetrix) reproducibility of this HTTr platform. Our results show that, while the read depth and fraction of probes with zero read count varied between samples (Fig. 1a, b), a strong correlation (mean correlation coefficient greater than 0.99) between read counts of technical replicates was observed (Fig. 1c). The finding is consistent with a previous study examining the reproducibility of TempO-Seq (Yeakley et al., 2017). Of note, technical replicates in our study design were three independent samples of RNAs extracted from primary rat hepatocytes; therefore, a strong correlation between technical replicates would indicate that the experimental steps, starting from cell seeding to library preparation, are highly reproducible. A strong correlation was also observed between read counts of biological replicates, albeit the mean correlation coefficient was lower than that of technical replicates, which was attributed to the additional source of variations arising from different cell source and separate sequencing runs (Fig. 1).

It is noteworthy that a strong correlation between replicates does not necessarily reflect agreement, as the large number of targeted genes, particularly in the WT assay, makes it difficult for linear relationships to be skewed by strong disagreement between a small number of genes (McIntyre et al., 2011). Nonetheless, based on the results and taking the cost of sequencing a large number of replicates into consideration, we believe that technical replicates are non-essential for the present HTTr platform but biological replicates are highly recommended.

Our results also showed that the reproducibility of DEGs between biological replicates positively correlates with the magnitude of transcriptional effect induced by the treatment, which depends on the potency of the chemical and the concentration (Fig. 2). A more reproducible gene signature is observed between biological replicates of hepatocytes treated with a chemical that induces several thousand DEGs than with one that induces only a few DEGs, or between high and low concentrations for the same chemical. Similar observations were reported in studies comparing the reproducibility of DEGs between samples from different sources. For instance, a study examining the concordance of drug-induced transcriptional response between rodent livers exposed to the same chemical but from different expression datasets showed that concordance in gene expression was highest for treatments causing the largest transcriptional effects (Sutherland et al., 2016). Also, a comparison between Affymetrix gene chips and RNA-Seq demonstrated that the cross-platform concordance in DEGs positively correlated with the degree of perturbation elicited by the treatment (C. Wang et al., 2014). Based on these findings, testing the highest non-cytotoxic chemical concentration would ensure the most reproducible gene signatures between replicates.

On the other hand, testing concentrations above the highest non-cytotoxic concentration should be avoided as it may not yield information relevant to the mode of action of a chemical (Waldmann et al., 2014). Accordingly, identifying the highest non-cytotoxic dose via cell viability screening is a necessary first step in designing an HTTr study. Moreover, since a gene can be differentially expressed in the opposite direction at different concentrations of the same chemical (Supplementary Fig. 3), measuring a range of concentrations below the highest non-cytotoxic concentration would yield useful information on dose-dependent transcriptomic responses.

Cross-platform reproducibility was evaluated by comparing TempO-Seq data with the Open TG-GATES data set using connectivity mapping (Lamb, 2007). We used the *in vitro* rat primary hepatocyte subset of the Open TG-GATES data, which was most closely aligned with our study design, including 131 chemicals a set of 1177 transcriptomic profiles. Using five different connectivity-scoring algorithms to quantify similarities between all TempO-Seq and Affymetrix transcriptomic data, we found 68% of the chemicals were perfectly matched between the two platforms and 100% of the chemicals were in the top 10 hits. Connectivity mapping from TempO-Seq ST and WT and the TG-GATES data showed a high level of concordance (Fig. 3b). Furthermore, our preliminary results suggest that similarity between TempO-Seq and TG-GATES transcriptomic profiles can be indicative of common mechanisms of action for chemicals. The high level of cross-platform reproducibility reinforces our findings on the quality of the TempO-Seq ST assay for characterizing the effects of chemicals on hepatocytes and its suitability for large-scale screening of environmental chemicals.

This study was also designed to evaluate the robustness of the S1500+ gene set as a surrogate to the whole transcriptome in representing transcriptional diversity and pathway coverage. While the comparison could be done by simply extracting the S1500+ genes and associated read counts from the WT assay, such an approach does not take into account the differences in read depth between the two assays (Fig. 1a). The two assays also differ in that there are 336 probes targeting the same genes but with different detector oligo sequences, which was a result of the continuous improvement in the probe design process. These inherent differences between the two assays could introduce variations in the differential gene expression. Thus, to perform an accurate comparison, two sequencing libraries were prepared for each sample, one for the S1500+ gene set (ST assay) and the other for the whole transcriptome (WT assay). Notwithstanding the differences between the two assays, an overall positive correlation in the L2FC values was observed (Supplementary Fig. 4). Notably, despite targeting a smaller number of genes, DEGs derived from ST assay were able to cluster treatments into groups similar to that of WT assay (Fig. 3). Our finding is in agreement with a previous report showing that the TempO-Seq ST assay is comparable to other whole transcriptome sequencing approaches (microarray and RNA-Seq) in driving the clustering of samples from liver of rats exposed to chemicals based on the mechanism of action of the chemicals (Bushel et al., 2018).

GSEA was performed to evaluate the ST assay in detecting chemical-induced changes in biological processes in comparison to the WT assay. Our results showed that the transcriptomic signatures acquired by ST assay could yield enriched gene sets relevant to the MOA of the drugs and consistent with WT assay. Nonetheless, the number of enriched gene sets was lower than WT assay when the gene sets were filtered by statistical significance (Fig. 5). When comparison was performed based on enrichment scores (NES), which reflects the degree to which the genes in a gene set are uniformly up- or down-regulated (Subramanian et al., 2005), a strong correlation between the two assays was observed (Supplementary Fig. 5). Clustering samples using NES calculated by ST and WT assays correctly paired most of the treatments, further demonstrating the consistency in gene set enrichment between the two assays (Fig. 6). These results indicated that the ST assay is capable of detecting chemical-induced perturbations in biological processes similar to that of WT assay.

GSEA was originally designed to run on the whole transcriptome, therefore running GSEA on ST datasets raises the concern of whether the size of a gene set after restricting to the S1500+ genes would be too small (or too large) for accurate results. According to the GSEA User Guide version 3.0, the recommended minimum number of genes in a gene set is 15. Seven hallmark gene sets had<15 genes in the S1500+ panel, namely, Notch signaling, Hedgehog signaling, pancreas beta cells, apical surface, WNT beta-catenin signaling, KRAS signaling DN, and angiogenesis (Supplementary Table 4). Also, the recommended maximum gene set size is 500 for datasets with 10,000 to 20,000 features, or between 2.5 and 5% of the expressed genes. mTORC1 signaling with 92 genes, or approximately 5.2% of the S1500+ genes after taking into account of gene isoforms, was found to exceed this range. Therefore, one should be mindful of these 8 hallmark gene sets when interpreting the enrichment scores of ST datasets.

A consistent pattern of gene set enrichment among chemicals associated with human DILI was identified—positive enrichment of apoptosis, cell proliferation, and inflammatory signaling pathways coupled with negative enrichment of metabolic and biosynthetic processes (Fig. 6). Several treatments were found to match this pattern of gene sets enrichment, but not all were identified as cytotoxic using CellTiter Glo assay, which corroborates previous findings that changes in gene expression occur prior to changes in cytotoxicity (Limonciel et al., 2018, Waldmann et al., 2014). The top 5 hallmark gene sets driving the clustering of treatments were known to be closely associated with hepatotoxicity (Supplementary Table 5). Apoptosis is a tightly regulated process that can be triggered by either the extrinsic pathway (binding of death receptors) or the intrinsic pathway (or mitochondrial pathway). One of the ways in which drugs and their reactive metabolites trigger apoptosis is by producing reactive oxygen species, causing mitochondrial damage that leads to the release of apoptogenic factors such as cytochrome *c* (Wang, 2014). Reactive metabolites could also cause DNA damage and activate the p53 pathway; for this reason, p53 is one of the key gene signatures investigated for routine monitoring of drug safety (Podtelezhnikov et al., 2020). Both apoptosis and the p53 pathway were positively enriched in hepatotoxic treatments (Supplementary Fig. 7). The liver synthesizes most of the coagulation factors, and coagulation impairment is associated with liver damage (Kopec and Luyendyk, 2014). The liver also plays a central role in xenobiotic and bile acid metabolisms, and disorders in hepatic metabolism are associated with liver diseases (Chiang, 2013). Hallmark gene sets for coagulation, bile acid metabolism and xenobiotic metabolism were negatively enriched in hepatotoxic treatments (Supplementary Fig. 7).

In this work, we combined a collagen sandwich model, S1500+ high-throughput transcriptomics and hallmark gene set enrichment as a resource-sparing and precise way of acquiring and analyzing transcriptomic data for *in vitro* assessment of hepatotoxicity. Moreover, clustering of NES of hallmark gene set provides a way of differentiating between hepatotoxic and non-hepatotoxic chemicals. This HTTr platform is amenable to scale up as both the collagen sandwich model and the TempO-Seq assay can be implemented in 384-well format with robotic automation; thus it also has the potential of screening thousands of chemicals at different dosing concentrations in one experiment.

## Funding

This work is supported in part by the Institute of Bioengineering and Bioimaging, Biomedical Research Council, Agency for Science, Technology and Research (A*STAR): A*STAR via IFCS (Project Number IAF111220, IAF311017B, IAF-PP H18/01/a0/014, IAF-PP H18/01/a0/K14); via EMULSION IAF (R-185-000-350-305); MOE ARC (R-185-000-342-112); SMART CAMP; and Mechanobiology Institute of Singapore (R-714-106-004-135) funding to Hanry Yu.

## CRediT authorship contribution statement

**Fan Lee:** Investigation, Formal analysis, Writing - original draft. **Imran Shah:** Funding acquisition, Supervision, Conceptualization, Formal analysis, Writing - review & editing. **Yun Ting Soong:** Investigation, Writing - review & editing. **Jiangwa Xing:** Conceptualization. **Inn Chuan Ng:** Resources. **Farah Tasnim:** Conceptualization, Funding acquisition, Project administration. **Hanry Yu:** Supervision, Funding acquisition, Writing - review & editing.

## Declaration of Competing Interest

Hanry Yu declares he holds significant equity in Invitrocue, Osteopore, Histoindex, Vasinfuse and Ants Innovate not related to the reported work. The authors declare they have no actual or potential competing financial interests.
