# Supplementary material for: Reproducibility and robustness of high-throughput S1500+ transcriptomics on primary rat hepatocytes for chemical-induced hepatotoxicity assessment
Source: Curr Res Toxicol. 2021 Aug 5;2:282–95. doi: 10.1016/j.crtox.2021.07.003 (PMC8384775; doi:10.1016/j.crtox.2021.07.003)
Supplement: Supplementary data 1 [file mmc1.docx]

Supplementary Material

Reproducibility and Robustness of High-Throughput S1500+ Transcriptomics on Primary Rat Hepatocytes for Chemical-Induced Hepatotoxicity Assessment

Fan Lee^1^, Imran Shah^2*^, Yun Ting Soong^1­^, Jiangwa Xing^1­^, Inn Chuan Ng^3^, Farah Tasnim^1^, and Hanry Yu^1,3,4*^

^1^ Innovations in Food & Chemical Safety Program (IFCS), Institute of Bioengineering and Nanotechnology (IBN), Agency for Science Technology and Research, Singapore.

^2^ Center for Computational Toxicology & Exposure, Office of Research and Development, U.S. Environmental Protection Agency, Research Triangle Park, North Carolina, USA

^3^ Department of Physiology and Mechanobiology Institute, National University of Singapore, Singapore

^4^ Critical Analytics for Manufacturing Personalized-Medicine, Singapore-MIT Alliance for Research and Technology, Singapore

***Address correspondence to**: Imran Shah (email: [Shah.Imran@epa.gov](mailto:Shah.Imran@epa.gov)) and Hanry Yu (email: [hyu@ibn.a-star.edu.sg](mailto:hyu@ibn.a-star.edu.sg))

**Supplementary Table 1.** Viability of primary rat hepatocytes after 24 h chemical exposure (n = 3, mean ± standard deviation).

| **Chemical** | **Concentration (µM)** | **Viability (%)** |
| --- | --- | --- |
| Acetaminophen | 400  10,000 | 109.2 ± 2.7  113.8 ± 2.3 |
| Caffeine | 400  10,000 | 102.4 ± 5.4  113.2 ± 1.9 |
| Chloramphenicol | 18  450 | 102.0 ± 4.4  105.0 ± 4.3 |
| Diclofenac | 16  400 | 102.5 ± 1.9  86.6 ± 5.4 |
| Doxorubicin | 0.08  2 | 102.9 ± 4.2  107.2 ± 4.1 |
| Indomethacin | 12  300 | 105.9 ± 2.9  78.1 ± 11.2 |
| Isoniazid | 400  10,000 | 108.4 ± 3.4  117.9 ± 2.6 |
| Ketoconazole | 0.6  15 | 108.3 ± 4.4  126.6 ± 4.4 |
| Naproxen | 80  2000 | 111.8 ± 2.0  113.4 ± 2.2 |
| Ranitidine | 160  4000 | 98.6 ± 4.1  102.3 ± 4.9 |
| Simvastatin | 2  60 | 102.8 ± 8.0  104.4 ± 5.7 |
| Sulfasalazine | 4  100 | 105.3 ± 1.8  115.7 ± 3.1 |
| Valproic acid | 400  10,000 | 107.7 ± 5.0  74.3 ± 1.1 |
| WY-14643 | 8  200 | 116.9 ± 6.4  93.1 ± 5.3 |

**Supplementary Table 2.** The entire connectivity mapping analysis data (separate file).

**Supplementary Table 3.** Summary of performance results for connectivity mapping. The mean and standard deviation (std) of performance scores calculated using the fraction of treatments where the best match is the hit (Fr1), where the hit is in the top five matches (Fr5) and the hit is in the top 10 matches (Fr10). Connectivity-scoring (CS) algorithms include: signed Jaccard index (sji), gene set total enrichment score (gtes)⁠, extreme cosine (xc) score⁠, Pearson correlation coefficient (xcp) and Spearman correlation coefficient (xcs).

|  |  | ST | | WT | |
| --- | --- | --- | --- | --- | --- |
|  |  | **Performance** | | **Performance** | |
|  |  | **mean** | **std** | **mean** | **std** |
| **Metric** | **CS** |  |  |  |  |
| **Fr1** | **gtes** | 0.11 | 0.02 | 0.20 | 0.03 |
|  | **sji** | 0.37 | 0.04 | 0.40 | 0.04 |
|  | **ts** | 0.03 | 0.04 | 0.10 | 0.01 |
|  | **xc** | 0.46 | 0.05 | 0.36 | 0.04 |
|  | **xcp** | 0.44 | 0.05 | 0.38 | 0.06 |
|  | **xcs** | 0.40 | 0.06 | 0.40 | 0.07 |
|  | **xm** | 0.18 | 0.06 | 0.21 | 0.02 |
| **Fr10** | **gtes** | 0.60 | 0.03 | 0.63 | 0.04 |
|  | **sji** | 0.68 | 0.04 | 0.74 | 0.04 |
|  | **ts** | 0.43 | 0.04 | 0.34 | 0.03 |
|  | **xc** | 0.84 | 0.03 | 0.82 | 0.03 |
|  | **xcp** | 0.83 | 0.04 | 0.79 | 0.02 |
|  | **xcs** | 0.73 | 0.05 | 0.77 | 0.03 |
|  | **xm** | 0.63 | 0.02 | 0.64 | 0.02 |
| **Fr5** | **gtes** | 0.53 | 0.01 | 0.50 | 0.05 |
|  | **sji** | 0.62 | 0.02 | 0.65 | 0.05 |
|  | **ts** | 0.33 | 0.03 | 0.25 | 0.02 |
|  | **xc** | 0.70 | 0.02 | 0.70 | 0.03 |
|  | **xcp** | 0.70 | 0.02 | 0.69 | 0.02 |
|  | **xcs** | 0.63 | 0.03 | 0.67 | 0.03 |
|  | **xm** | 0.58 | 0.02 | 0.58 | 0.02 |

**Supplementary Table 4.** Size of hallmark gene sets before and after restricting to the genes in ST or WT assays.​

| **Hallmark Gene Sets** | **Original**  **Size** | **Restricting to ST Assay** | **Restricting to WT Assay** |
| --- | --- | --- | --- |
| TNFA SIGNALING VIA NFKB | 200 | 69 | 146 |
| HYPOXIA | 200 | 75 | 143 |
| CHOLESTEROL HOMEOSTASIS | 74 | 36 | 57 |
| MITOTIC SPINDLE | 199 | 34 | 160 |
| WNT BETA CATENIN SIGNALING | 42 | 11 | 22 |
| TGF BETA SIGNALING | 54 | 16 | 46 |
| IL6 JAK STAT3 SIGNALING | 87 | 20 | 52 |
| DNA REPAIR | 150 | 30 | 114 |
| G2M CHECKPOINT | 200 | 63 | 155 |
| APOPTOSIS | 161 | 76 | 110 |
| NOTCH SIGNALING | 32 | 5 | 22 |
| ADIPOGENESIS | 200 | 64 | 158 |
| ESTROGEN RESPONSE EARLY | 200 | 45 | 125 |
| ESTROGEN RESPONSE LATE | 200 | 56 | 118 |
| ANDROGEN RESPONSE | 100 | 32 | 75 |
| MYOGENESIS | 200 | 40 | 91 |
| PROTEIN SECRETION | 96 | 22 | 82 |
| INTERFERON ALPHA RESPONSE | 97 | 27 | 60 |
| INTERFERON GAMMA RESPONSE | 200 | 56 | 113 |
| APICAL JUNCTION | 200 | 37 | 110 |
| APICAL SURFACE | 44 | 9 | 20 |
| HEDGEHOG SIGNALING | 36 | 7 | 17 |
| COMPLEMENT | 200 | 66 | 122 |
| UNFOLDED PROTEIN RESPONSE | 113 | 46 | 96 |
| PI3K AKT MTOR SIGNALING | 105 | 44 | 78 |
| MTORC1 SIGNALING | 200 | 92 | 162 |
| E2F TARGETS | 200 | 81 | 155 |
| MYC TARGETS V1 | 200 | 52 | 163 |
| MYC TARGETS V2 | 58 | 25 | 50 |
| EPITHELIAL MESENCHYMAL TRANSITION | 200 | 60 | 97 |
| INFLAMMATORY RESPONSE | 200 | 38 | 95 |
| XENOBIOTIC METABOLISM | 200 | 84 | 160 |
| FATTY ACID METABOLISM | 158 | 72 | 118 |
| OXIDATIVE PHOSPHORYLATION | 200 | 39 | 170 |
| GLYCOLYSIS | 200 | 58 | 140 |
| REACTIVE OXYGEN SPECIES PATHWAY | 49 | 24 | 40 |
| P53 PATHWAY | 200 | 63 | 143 |
| UV RESPONSE UP | 158 | 55 | 115 |
| UV RESPONSE DN | 144 | 41 | 97 |
| ANGIOGENESIS | 36 | 13 | 20 |
| HEME METABOLISM | 200 | 34 | 125 |
| COAGULATION | 138 | 53 | 96 |
| IL2 STAT5 SIGNALING | 200 | 42 | 119 |
| BILE ACID METABOLISM | 112 | 31 | 82 |
| PEROXISOME | 104 | 36 | 79 |
| ALLOGRAFT REJECTION | 200 | 37 | 80 |
| SPERMATOGENESIS | 135 | 17 | 45 |
| KRAS SIGNALING UP | 200 | 33 | 106 |
| KRAS SIGNALING DN | 200 | 11 | 55 |
| PANCREAS BETA CELLS | 40 | 7 | 12 |

**Supplementary Table 5.** t-test of hallmark gene sets NES between samples in Cluster A (non-hepatotoxic) and B (hepatotoxic) of Supplementary Figure 5**.** Gene sets were ranked by ascending order of FDR.

| **No.** | **Hallmark Gene Sets** | **Mean NES of Cluster A** | **Mean NES of Cluster B** | **Abs. difference between the means** | **FDR** |
| --- | --- | --- | --- | --- | --- |
| 1 | XENOBIOTIC METABOLISM (MET) | -1.72 | 1.70 | 3.41 | 6.31E-10 |
| 2 | BILE ACID METABOLISM (MET) | -1.59 | 1.95 | 3.55 | 1.39E-08 |
| 3 | APOPTOSIS (PAT) | 1.27 | -1.47 | 2.74 | 1.10E-07 |
| 4 | P53 PATHWAY (PRO) | 1.54 | -1.17 | 2.71 | 1.73E-06 |
| 5 | COAGULATION (IMM) | -2.05 | 0.60 | 2.65 | 2.35E-04 |
| 6 | PI3K AKT MTOR SIGNALING (SIG) | 1.50 | -0.30 | 1.80 | 5.87E-04 |
| 7 | WNT BETA CATENIN SIGNALING (SIG) | 0.65 | -1.51 | 2.15 | 1.34E-03 |
| 8 | MYC TARGETS V1 (PRO) | 1.54 | -0.32 | 1.86 | 1.37E-03 |
| 9 | DNA REPAIR (DD) | 1.62 | 0.28 | 1.34 | 1.40E-03 |
| 10 | TNFA SIGNALING VIA NFKB (SIG) | 0.79 | -1.78 | 2.56 | 5.15E-03 |
| 11 | TGF BETA SIGNALING (SIG) | 0.51 | -1.57 | 2.08 | 6.05E-03 |
| 12 | INFLAMMATORY RESPONSE (IMM) | 0.57 | -1.56 | 2.13 | 6.97E-03 |
| 13 | INTERFERON GAMMA RESPONSE (IMM) | -0.06 | -1.79 | 1.73 | 1.24E-02 |
| 14 | FATTY ACID METABOLISM (MET) | -0.26 | 2.23 | 2.49 | 1.34E-02 |
| 15 | ADIPOGENESIS (DEV) | -0.08 | 1.99 | 2.07 | 1.42E-02 |
| 16 | PEROXISOME (CC) | -0.04 | 2.01 | 2.04 | 1.42E-02 |
| 17 | INTERFERON ALPHA RESPONSE (IMM) | -0.88 | -1.96 | 1.08 | 2.00E-02 |
| 18 | OXIDATIVE PHOSPHORYLATION (MET) | -0.06 | 1.85 | 1.91 | 2.00E-02 |
| 19 | EPITHELIAL MESENCHYMAL TRANSITION (DEV) | 0.53 | -1.03 | 1.56 | 2.37E-02 |
| 20 | APICAL JUNCTION (CC) | 0.29 | -1.32 | 1.62 | 2.77E-02 |
| 21 | HEDGEHOG SIGNALING (SIG) | -0.41 | 0.84 | 1.25 | 2.77E-02 |
| 22 | MYC TARGETS V2 (PRO) | 1.13 | -0.32 | 1.45 | 2.77E-02 |
| 23 | HEME METABOLISM (MET) | -0.05 | 1.10 | 1.15 | 2.77E-02 |
| 24 | ALLOGRAFT REJECTION (IMM) | 0.12 | -1.30 | 1.42 | 2.77E-02 |
| 25 | IL2 STAT5 SIGNALING (SIG) | 0.72 | -0.68 | 1.40 | 3.30E-02 |
| 26 | KRAS SIGNALING DN (SIG) | -1.60 | -0.99 | 0.61 | 4.50E-02 |
| 27 | UV RESPONSE UP (DD) | 0.73 | -0.43 | 1.16 | 4.51E-02 |
| 28 | SPERMATOGENESIS (DEV) | -0.13 | 0.95 | 1.08 | 4.77E-02 |
| 29 | APICAL SURFACE (CC) | 0.10 | -0.97 | 1.07 | 7.22E-02 |
| 30 | IL6 JAK STAT3 SIGNALING (IMM) | 0.21 | -0.84 | 1.05 | 7.42E-02 |
| 31 | REACTIVE OXYGEN SPECIES PATHWAY (PAT) | 0.09 | 1.07 | 0.98 | 9.32E-02 |
| 32 | ESTROGEN RESPONSE LATE (SIG) | -0.06 | 1.15 | 1.21 | 9.41E-02 |
| 33 | GLYCOLYSIS (MET) | -0.17 | 0.87 | 1.04 | 1.03E-01 |
| 34 | G2M CHECKPOINT (PRO) | 1.09 | 0.25 | 0.84 | 1.10E-01 |
| 35 | E2F TARGETS (PRO) | 1.40 | 0.60 | 0.79 | 1.34E-01 |
| 36 | MTORC1 SIGNALING (SIG) | 1.72 | 1.32 | 0.40 | 1.41E-01 |
| 37 | COMPLEMENT (IMM) | -1.01 | -0.21 | 0.80 | 1.45E-01 |
| 38 | MYOGENESIS (DEV) | 0.92 | 0.23 | 0.69 | 2.00E-01 |
| 39 | UV RESPONSE DN (DD) | 0.07 | -0.76 | 0.83 | 2.36E-01 |
| 40 | ESTROGEN RESPONSE EARLY (SIG) | 0.72 | 1.26 | 0.54 | 2.94E-01 |
| 41 | ANGIOGENESIS (DEV) | -0.87 | -0.25 | 0.62 | 2.94E-01 |
| 42 | HYPOXIA (PAT) | 0.58 | 1.10 | 0.52 | 3.06E-01 |
| 43 | ANDROGEN RESPONSE (SIG) | 1.31 | 0.80 | 0.51 | 3.46E-01 |
| 44 | KRAS SIGNALING UP (SIG) | -0.66 | -0.08 | 0.58 | 3.84E-01 |
| 45 | CHOLESTEROL HOMEOSTASIS (MET) | 1.27 | 1.53 | 0.27 | 6.51E-01 |
| 46 | MITOTIC SPINDLE (PRO) | 0.04 | -0.17 | 0.20 | 7.81E-01 |
| 47 | PROTEIN SECRETION (PAT) | 0.03 | 0.18 | 0.15 | 7.81E-01 |
| 48 | NOTCH SIGNALING (SIG) | -0.75 | -0.83 | 0.08 | 8.40E-01 |
| 49 | UNFOLDED PROTEIN RESPONSE (PAT) | 0.74 | 0.62 | 0.11 | 8.40E-01 |
| 50 | PANCREAS BETA CELLS (DEV) | -0.94 | -0.84 | 0.10 | 8.40E-01 |


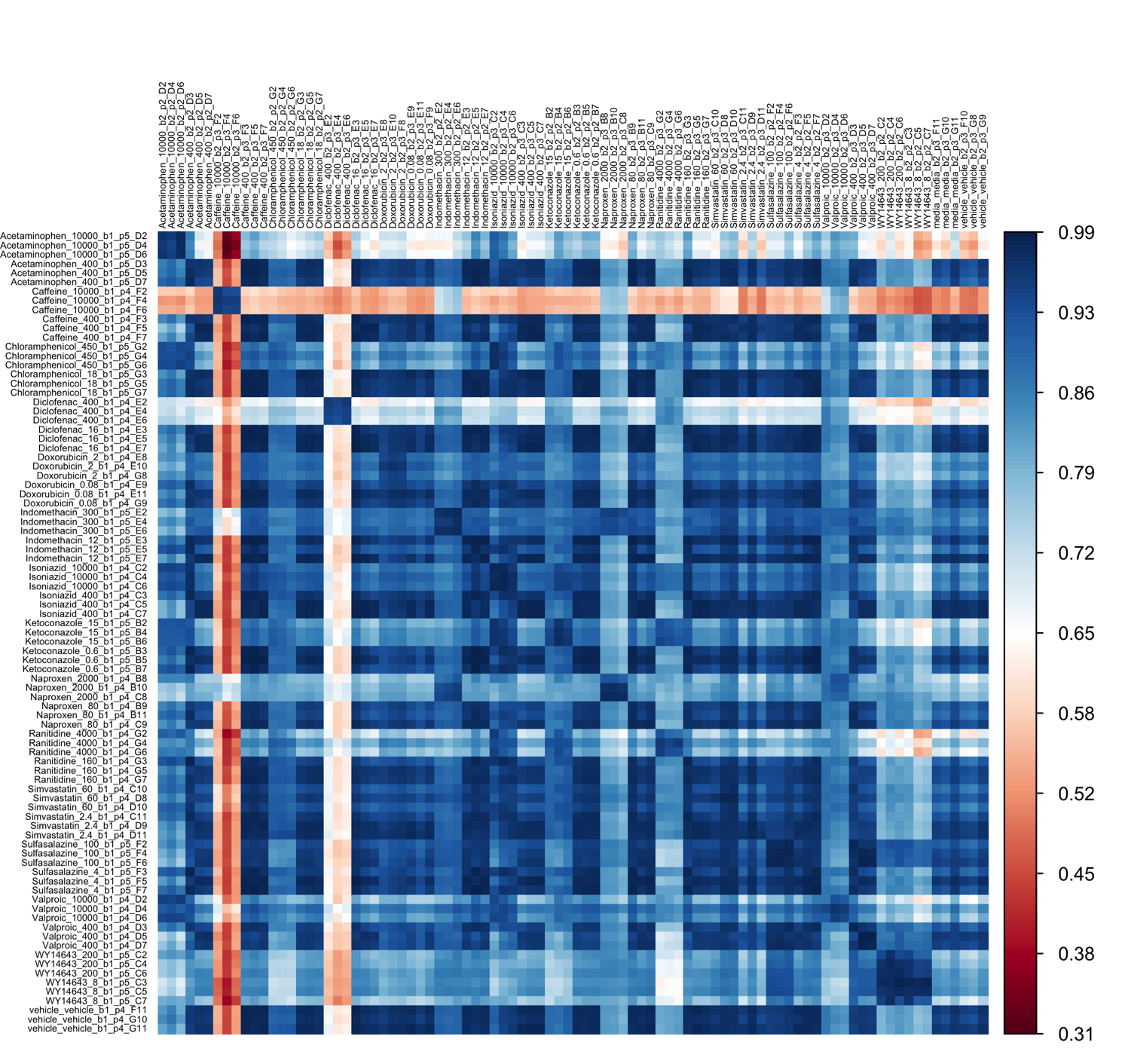


**Supplementary Figure 1.** Correlation coefficients of read counts of all samples in batch 1 and 2 against each other (ST assay). Strong correlations were observed between read counts of the same chemical treatment, i.e., biological replicates, for most treatments. Between different chemicals, stronger correlations were typically observed between low concentration samples than high concentration samples. This figure was generated by corrplot R package and the minimum (0.31) and maximum (0.99) correlation coefficients of the entire correlation matrix were designated as the lower and upper limit for the color of the correlogram. Sample label: chemical_concentration_batch_plate _well.


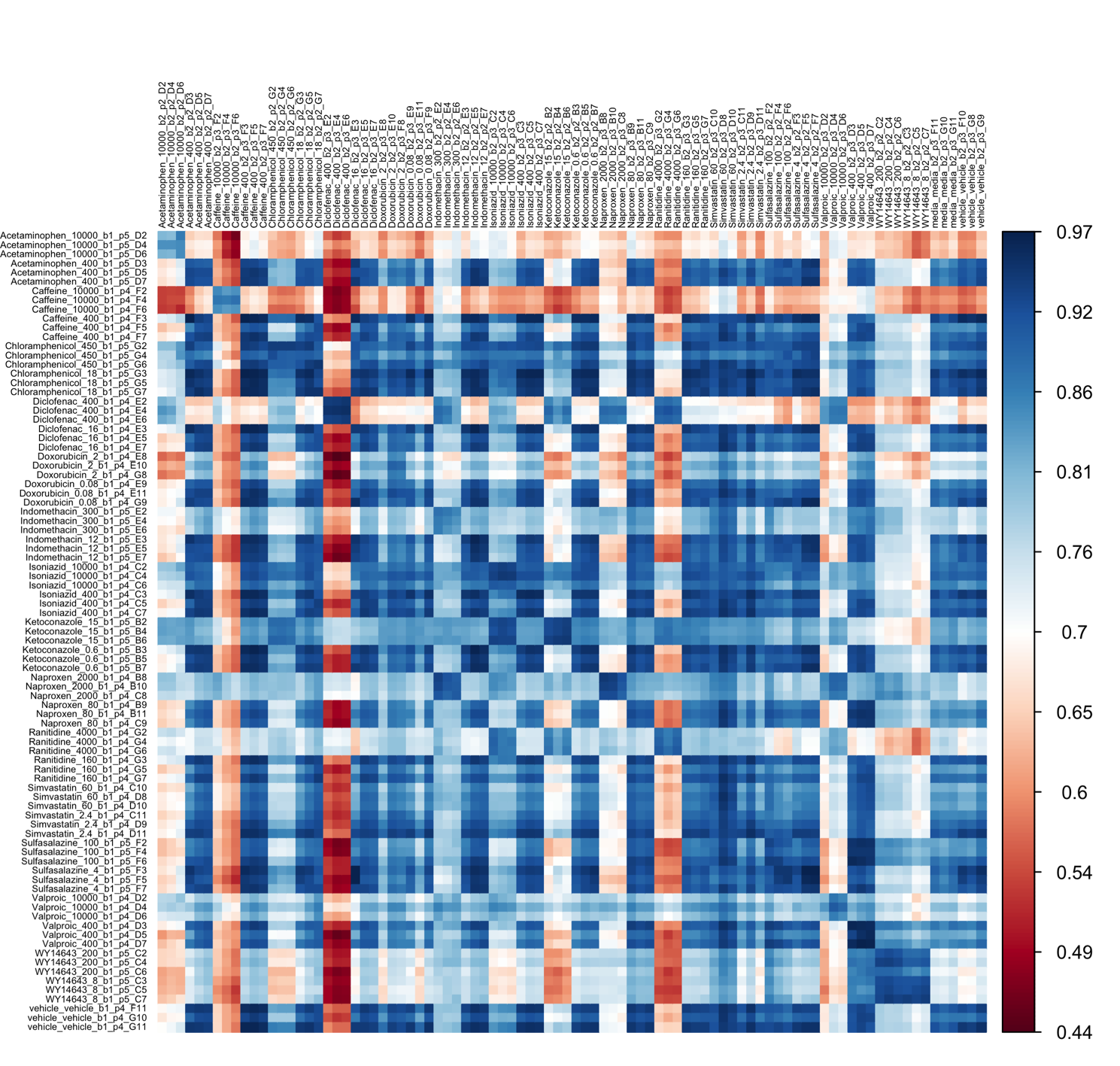


**Supplementary Figure 2.** Correlation coefficients of read counts of all samples in batch 1 and 2 against each other (WT assay). This figure was generated by corrplot R package and the minimum (0.44) and maximum (0.97) correlation coefficients of the entire correlation matrix were designated as the lower and upper limit for the color of the correlogram. Sample label: chemical_concentration_batch_plate _well.


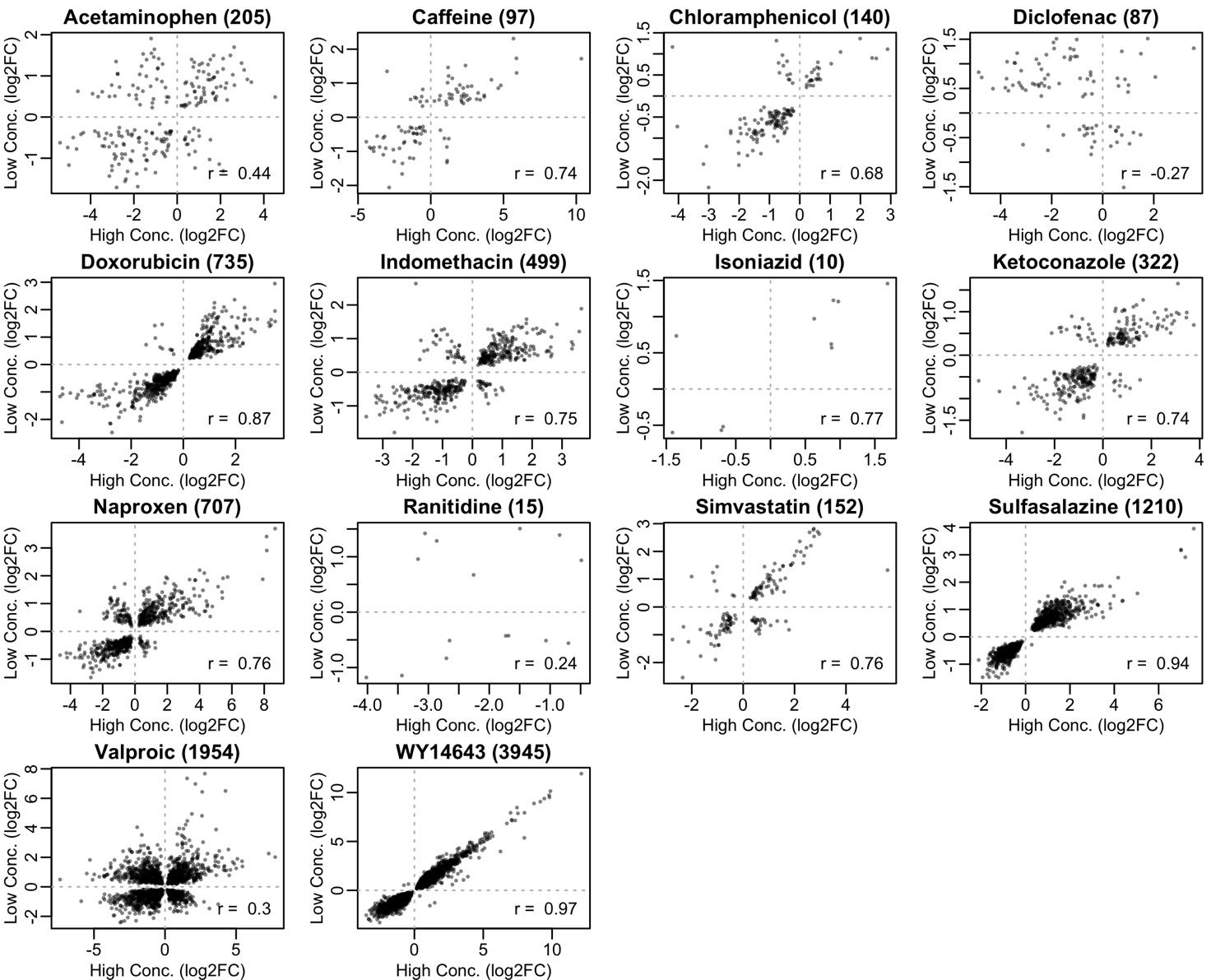


**Supplementary Figure 3.** The direction of gene expression depends on drug concentration. L2FC values of genes that were identified as differentially expressed (adjusted *p* < 0.05) at both low (Y-axis) and high (X-axis) concentrations were shown (WT assay of Batch 2). The number of genes identified as differentially expressed at both concentrations is shown in brackets next to the chemical name and the Pearson correlation coefficients of the L2FC values are shown within each plot.


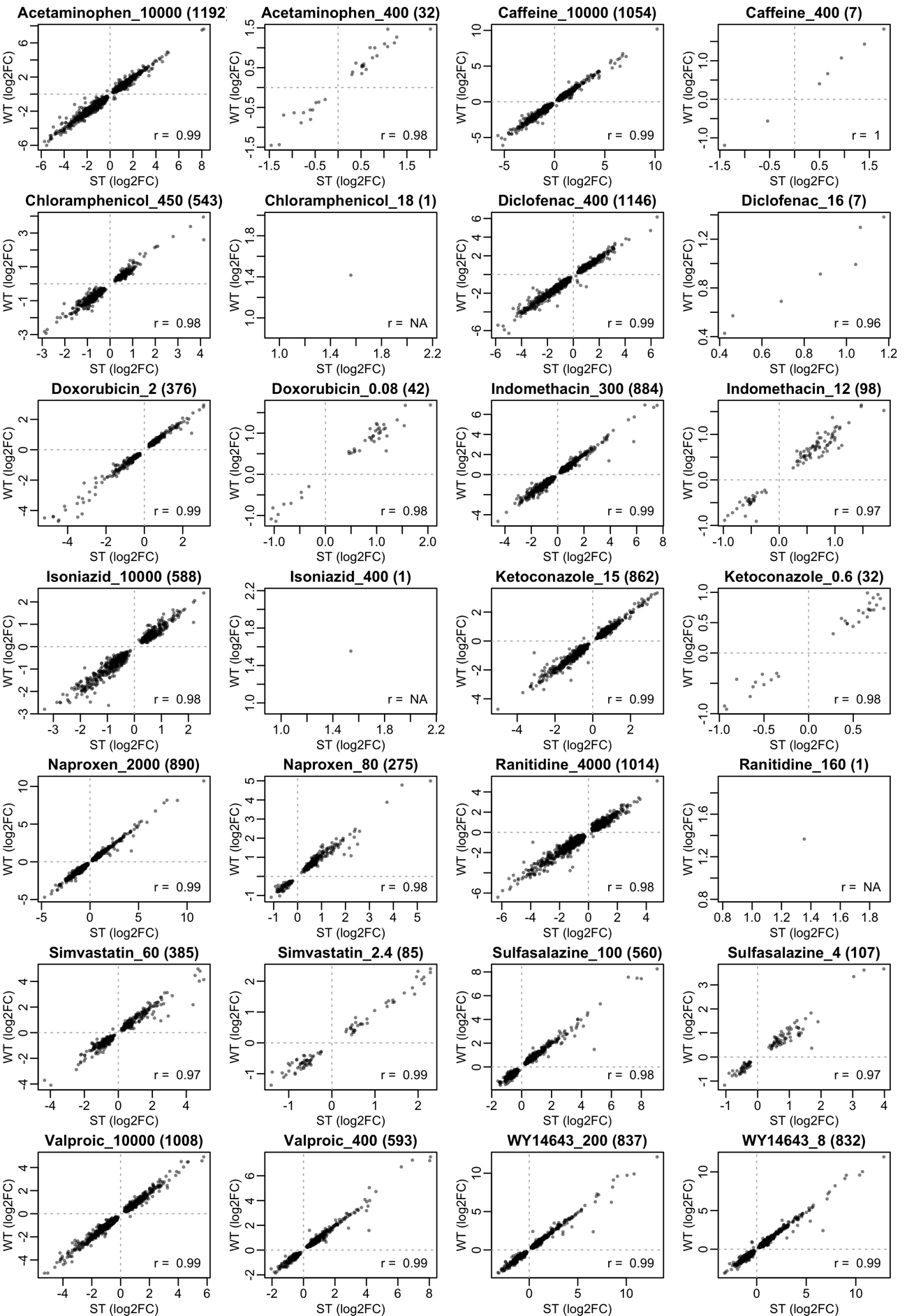


**Supplementary Figure 4.** Positive correlation of L2FC values of overlapping DEGs (adjusted *p* < 0.05) between ST (X-axis) and WT (Y-axis) assays. The number of overlapping DEGs is shown in brackets next to the treatment label. Pearson correlation coefficients of the L2FC values are shown within each plot.





**Supplementary Figure 5.** Comparison of NES between ST and WT assays. (a) Number of overlapping top 5 hallmark gene set ranked by descending order of NES between the two assays. (b) Pearson correlation coefficients of NES between the two assays.

**Supplementary Figure 6.** Unsupervised hierarchal clustering using NES of hallmark gene sets for ST samples alone.

**Supplementary Figure 7.** Unsupervised hierarchal clustering using NES of apoptosis, p53 pathway, coagulation, bile acids metabolism and xenobiotic metabolism.
